# Supplementary material for: A trefoil knot self-templated through imination in water
Source: Nat Commun. 2022 Jun 21;13:3557. doi: 10.1038/s41467-022-31289-1 (PMC9213439; doi:10.1038/s41467-022-31289-1)
Supplement: Supplementary file 1 — Supplementary Information final JSP [file 41467_2022_31289_MOESM1_ESM.pdf]

A trefoil knot self-templated through imination in water

Ye Lei[1,6], Zhaoyong Li[1,2,6], Guangcheng Wu[1], Lijie Zhang[3], Lu Tong[1], Tianyi Tong[4], Qiong Chen[1], Lingxiang Wang[1], Chenqi Ge[1], Yuxi Wei[1], Yuanjiang Pan[1], Andrew C.-H. Sue[4*], Linjun Wang[1,2*], Feihe Huang[1,5*], and Hao Li[1,5*]

[1]Department of Chemistry, Zhejiang University, Hangzhou 310027, PR China. [2]Key Laboratory of Excited-State Materials of Zhejiang Province, Zhejiang University, Hangzhou 310027, PR China. [3]Hangzhou Institute of Advanced Studies, Zhejiang Normal University, Hangzhou 311231, PR China. [4]College of Chemistry and Chemical Engineering, Xiamen University, Xiamen 361005, PR China. [5]ZJU-Hangzhou Global Scientific and Technological Innovation Center, Hangzhou 310027, PR China. [6]These authors contributed equally: Ye Lei, Zhaoyong Li. *Email: Andrewsue@xmu.edu.cn; ljwang@zju.edu.cn; fhuang@zju.edu.cn; lihao2015@zju.edu.cn

Supplementary Information

Table of Content

Supplementary Methods

1. Materials and general methods S3

2. Synthetic procedures S3–S7

3. Characterization S7–S20

4. Investigations of the self-assembly process S20–S28

5. Theoretical calculations S28–S29

Supplementary References S29

Supplementary Methods

1. Materials and general methods

All reagents and solvents were purchased from commercial sources and used without further purification. Nuclear magnetic resonance (NMR) spectra were recorded at ambient temperature using Bruker AVANCE III 400/500 or Agilent DD2 600 spectrometers, with working frequencies of 400/500/600 and 100/125/150 for ^1H and ^{13}C respectively. Chemical shifts are reported in ppm relative to the residual internal non-deuterated solvent signals (for proton NMR, D_2O: δ = 4.70 ppm, CD_3SOCD_3: δ = 2.50 ppm). High-resolution mass spectra (HRMS) were recorded on a Fourier transform ion cyclotron resonance mass spectrometry (FT-ICR MS). CD spectra were recorded on a Circular Dichroism Spectrometer (Chirascan V100, Applied Photophysics Ltd).

2. Synthetic procedures

The precursor **S1** was synthesized according to the procedure from the literature[1]. The procedure is decribed as follows. 5-bromoisophthalaldehyde (3.00 g, 14.08 mmol) and pyridine-4-boronic acid (2.08 g, 16.90 mmol) were dissolved in a mixture of THF (180 mL) and 2 M aqueous potassium carbonate (42.2 mL, 84.5 mmol).The mixture was then degassed (N_2 bubbling, 30 min) before the addition of $Pd(PPh_3)_4$ (325.2 mg, 0.28 mmol). The reaction was heated at 80 °C for 24 h. After cooling down the reaction solution to room temperature, the solvent was removed in vacuo. Purification via column chromatography (5:1 EtOAc/hexane) afforded the desired product **S1** as a white solid (1.51g, 7.12 mmol, 50%).

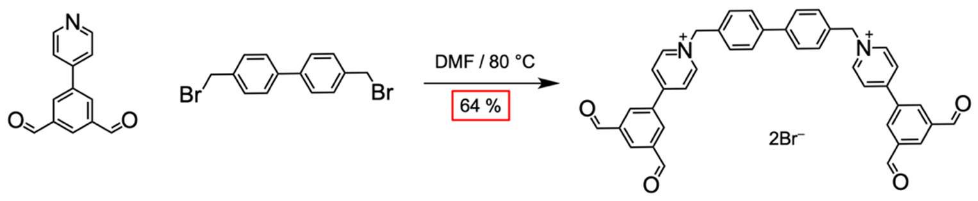

S$_1$ **1**$^{2+}$•2Br$^-$

Supplementary Scheme 1. Synthesis of 1^{2+}·2Br$^-$. 1-(Bromomethyl)-4-[4-(bromomethyl)phenyl]benzene (100 mg, 0.29 mmol) and **S**$_1$ (253mg, 1.2 mmol) were dissolved in DMF (15 mL). The corresponding reaction mixture was stirred at 80 °C overnight. After cooling down the reaction solution to room temperature, the yellow precipitate was collected by filtration and washed with CH_2Cl_2 for three times, yielding **1**$^{2+}$·2Br$^-$ (141 mg, 64%) as a yellow solid.

1^{2+}·2PF_6^- was obtained by performing counterion exchange to **1**$^{2+}$·2Br$^-$: **1**$^{2+}$·2Br$^-$ was dissolved in water at 90 °C, followed by adding NH_4PF_6 (100 mg), after which a white solid was collected by filtration and washed with water for three times. After drying the solid under vacuum, the pure compound **1**$^{2+}$·2PF_6^- was obtained in close to quantitative yield.

1^{2+}·2CH_3COO^- was obtained by performing counterion exchange to **1**$^{2+}$·2PF_6^-: **1**$^{2+}$·2PF_6^- was dissolved in CH_3CN, followed by adding TBA^+·CH_3COO^-, after which an olive green solid was collected by filtration and washed with CH_2Cl_2 for three times. After drying the solid under vacuum, **1**$^{2+}$·2CH_3COO^- was obtained in close to quantitative yield.

1^{2+}·2Br$^-$ or **1**$^{2+}$·2CH_3COO^- was characterized by NMR spectroscopy (Supplementary Figures 1–5). ^{1}H NMR (400 MHz, CD_3SOCD_3, 298 K) δ (ppm): 10.23 (s, 4H), 9.46 (d, *J*=8.0 Hz, 4H), 8.87 (d, *J*=1.2 Hz, 4H), 8.76 (d, *J*=8.0 Hz, 4H), 8.65 (t, *J*=1.2 Hz, 2H), 7.79 (d, *J*=8.0 Hz, 4H), 7.73 (d, *J*=8.0 Hz, 4H), 5.97 (s, 4H). ^{13}C NMR (100 MHz, CD_3SOCD_3, 298 K) δ (ppm): 192.2, 153.3, 145.3, 140.3, 137.8, 135.7, 134.2, 134.1, 132.5, 129.7, 127.7, 126.0, 62.4. HRMS: *m/z* calculated for $C_{40}H_{30}N_2O_4^{2+}$ ([**1**]$^{2+}$): 301.1097; found: 301.1113.

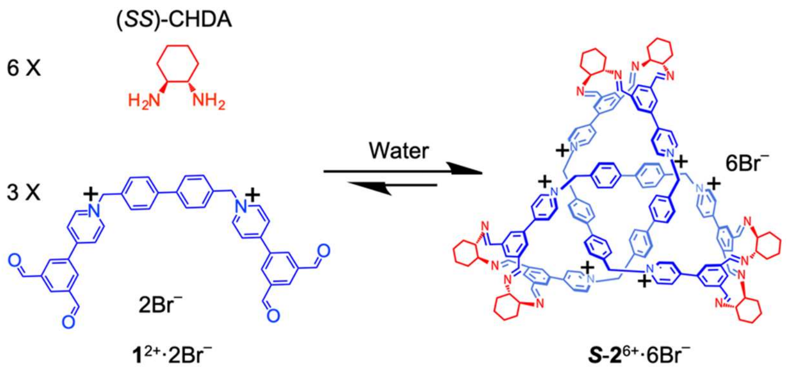

Supplementary Scheme 2. Synthesis of *S*-2^{6+}·6Br^{-}. A 1:2 mixture of **1**$^{2+}$·2Br^{-} (1.5 mg, 0.002 mmol) and (1*S*,2*S*)-(+)-1,2-diaminocyclohexane (0.46 mg, 0.004 mmol) was dissolved in D_2O (0.6 mL). The corresponding reaction mixtures were stirred at 80 °C for 12 h. After cooling down the reaction solution to room temperature, ^{1}H NMR (Supplementary Figure 24a) and MS (Supplementary Figure 14) spectra were recorded, indicating the success of self-assembly of the trefoil knot ***S*-2**$^{6+}$.

1^{2+}·2Br^{-} has a relatively low solubility in water. In order to obtain a solution of ***S*-2**$^{6+}$·6Br^{-} with a relatively high concentration, we sometimes preformed self-assembly of the trefoil knot in organic-water mixture, including either D_2O/CD_3SOCD_3 (3:1 v/v) or H_2O/CD_3SOCD_3 (3:1 v/v). The latter solvent helps to avoid deuteration.

NMR spectroscopic results of ***S*-2**$^{6+}$·6Br^{-} were shown in Supplementary Figures 6–13 and mass spectrum was shown in Supplementary Figure 14. ^{1}H NMR (600 MHz, D_2O/CD_3SOCD_3 (3:1 v/v), 298 K) δ (ppm): 8.61 (d, *J*=6.0 Hz, 12H), 8.45 (s, 12H), 8.04 (s, 6H), 7.99 (s, 6H), 7.91 (d, *J*=6.0 Hz, 12H), 7.46 (s, 6H), 6.76 (d, *J*=12.0 Hz, 12H), 5.72 (d, *J*=12.0 Hz, 6H), 5.65 (d, *J*=12.0 Hz, 6H), 5.42 (d, *J*=12.0 Hz, 12H), 3.62 (m, 6H), 3.33 (m, 6H), 1.04-2.10 (m, 48H). ^{13}C NMR (150 MHz, D_2O/CD_3SOCD_3 (3:1 v/v), 298 K) δ (ppm): 165.0, 162.4, 154.9, 145.5, 140.5, 138.8, 138.5, 136.1, 134.7, 133.7, 132.9, 129.5, 127.6, 127.2, 125.4, 76.6, 75.1, 64.0, 32.9, 32.4, 25.3. HRMS: For *S*-**2**$^{6+}$·6Br^{-}, *m/z* calculated for $C_{156}H_{149}N_{18}^{5+}$ ([**2**-H]$^{5+}$): 455.0444; found: 455.0437; *m/z* calculated for $C_{156}H_{150}N_{18}Br^{5+}$ ([**2**+Br]$^{5+}$): 471.4292; found: 471.4271; *m/z* calculated for $C_{156}H_{150}N_{18}Br_2^{4+}$ ([**2**+2Br]$^{4+}$): 609.2671; found: 609.2648.

When we use (*RR*)-CHDA or racemic CHDA instead of (*SS*)-CHDA, ***R*-2**$^{6+}$·6Br^{-} or a racemic mixture of ***R*-2**$^{6+}$·6Br^{-} and ***S*-2**$^{6+}$·6Br^{-} were obtained, respectively. The corresponding ^{1}H NMR spectra of ***R*-2**$^{6+}$·6Br^{-} and ***S*-2**$^{6+}$·6Br^{-}, as well as the racemic mixture were observed almost identical. The observation that racemic CHDA produced an identical spectrum implied the occurrence of narcissistic self-sorting. The CD spectra of ***R*-2**$^{6+}$, ***S*-2**$^{6+}$ showed mirror images while the racemic product is CD silent (Supplementary Figure 16).

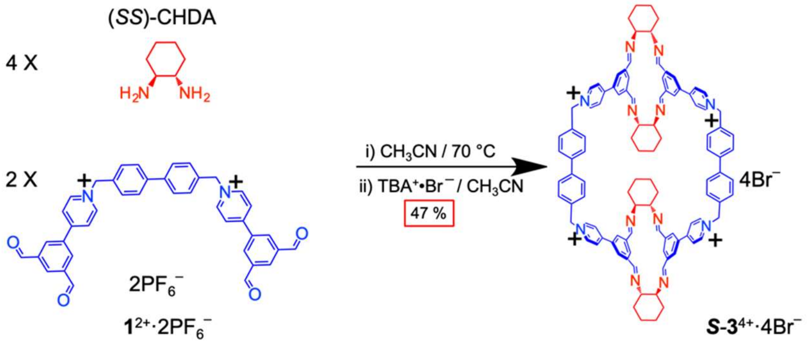

Supplementary Scheme 3. Synthesis of ***S*-3**$^{4+}$·4Br^{-}. A 1:2 mixture of **1**$^{2+}$·2PF$_6^{-}$ (30 mg, 0.034 mmol) and (1*S*,2*S*)-(+)-1,2-diaminocyclohexane (7.8 mg, 0.068 mmol) was dissolved in CH_3CN (18 mL). The corresponding reaction mixtures were stirred at 70 °C for 12 h. After cooling down the reaction solution to room temperature, tetrabutylammonium bromide (TBA^{+}·Br^{-}) (100 mg) was then added into the solution, after which a white solid was collected by filtration and washed with CH_2Cl_2 for three times, yielding the major compound ***S*-3**$^{4+}$·4Br^{-} (30 mg, 47%). It is noteworthy that raising the concentrations of the precursors namely **1**$^{2+}$·2PF$_6^{-}$ and (1*S*,2*S*)-(+)-1,2-diaminocyclohexane would lead to the formation of oligomeric and polymeric byproducts. ***S*-3**$^{4+}$·4Br^{-} was characterized by NMR spectroscopy (Supplementary Figures 17–21) and mass spectrum (Supplementary Figure 22). ^{1}H NMR (600 MHz, CD_3SOCD_3, 298 K) δ (ppm): 9.24 (d, *J*=6.0 Hz, 8H), 8.71 (s, 4H), 8.51 (d, *J*=6.0 Hz, 8H), 8.33 (s, 4H), 8.21 (s, 4H), 8.16 (s, 4H), 8.09 (s, 4H), 7.70 (d, *J*=12.0 Hz, 8H), 7.59 (d, *J*=12.0 Hz, 8H), 5.86-5.92 (m, 8H), 3.59-3.69 (m, 4H), 3.08-3.18 (m, 4H), 1.40-2.00 (m, 32H). ^{13}C NMR (100 MHz, CD_3SOCD_3, 298 K) δ (ppm): 161.9, 158.7, 154.5, 145.0,

140.2, 138.0, 137.8, 134.9, 134.1, 131.2, 130.9, 129.3, 127.6, 125.9, 125.7, 75.3, 71.4, 62.2, 32.7, 31.6, 24.2, 24.1. HRMS: For *S*-**3**$^{4+}$·4Br^{-}, *m/z* calculated for $C_{104}H_{100}N_{12}^{4+}$ ([**3**]$^{4+}$): 379.4551; found: 379.4520; *m/z* calculated for $C_{104}H_{99}N_{12}^{3+}$ ([**3**–H]$^{3+}$): 505.6044; found: 505.6017.

The CD spectra of ***S*-3**$^{4+}$·4Br^{-} and ***R*-3**$^{4+}$·4Br^{-} were also recorded (Supplementary Figure 23).

3. Characterization

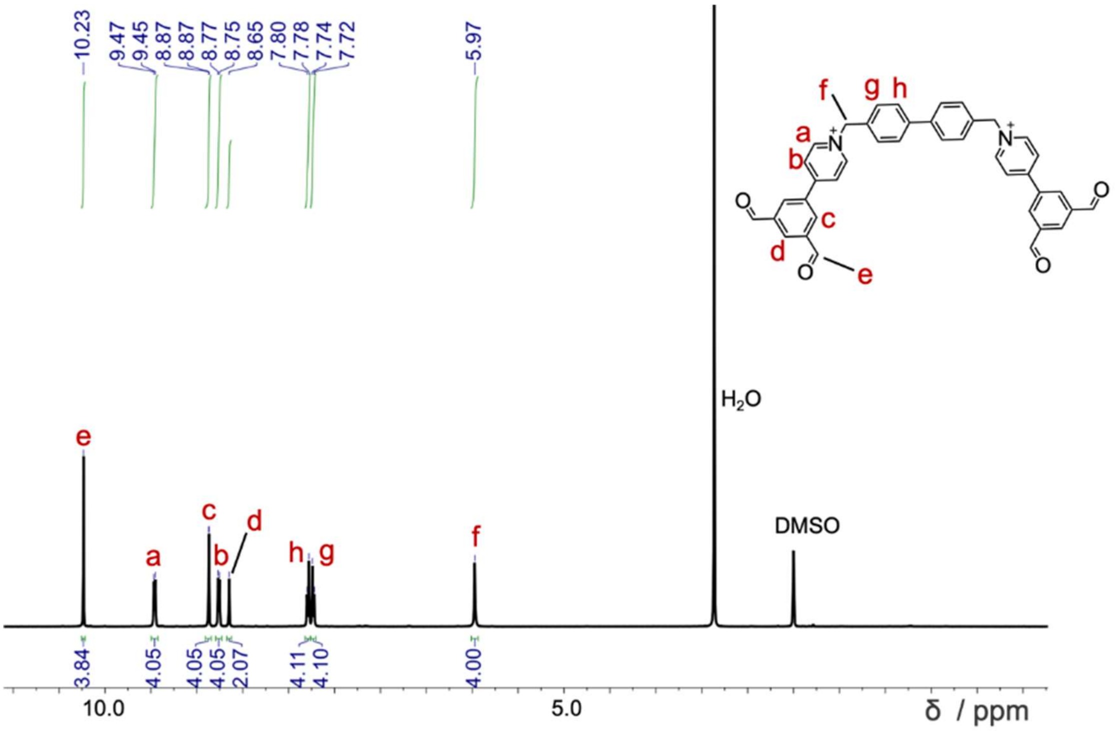

Supplementary Figure 1. NMR characterization. ^{1}H NMR spectrum (400 MHz, CD_3SOCD_3, 298 K) of **1**$^{2+}$·2Br^{-}.

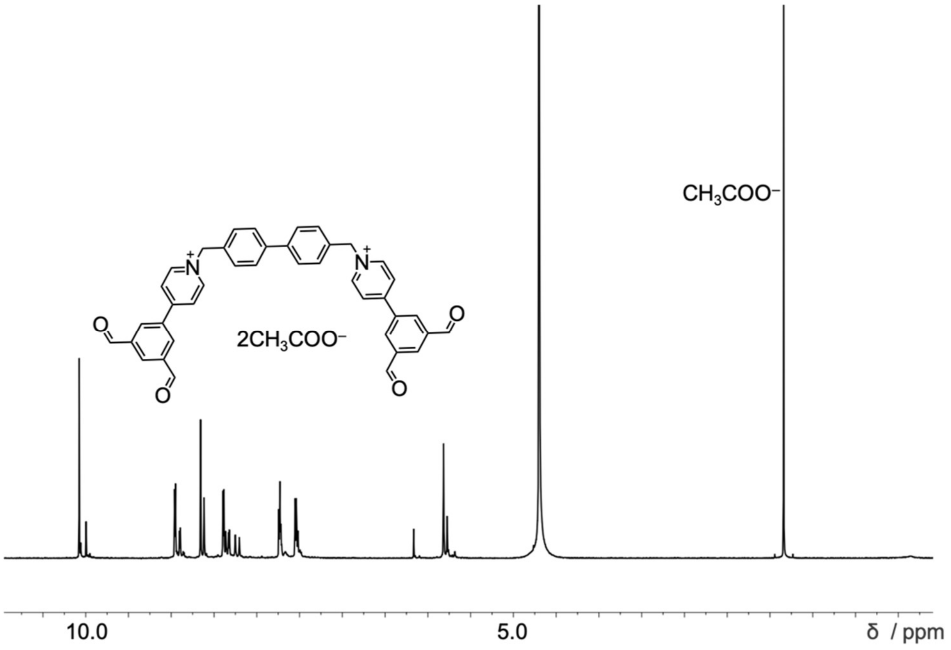

Supplementary Figure 2. NMR characterization. ^{1}H NMR spectrum (600 MHz, D_2O, 298 K) of partially hydrated $\mathbf{1}^{2+}\cdot 2CH_3COO^-$. $\mathbf{1}^{2+}\cdot 2CH_3COO^-$ would undergo decomposition partially due to the basicity of CH_3COO^-.

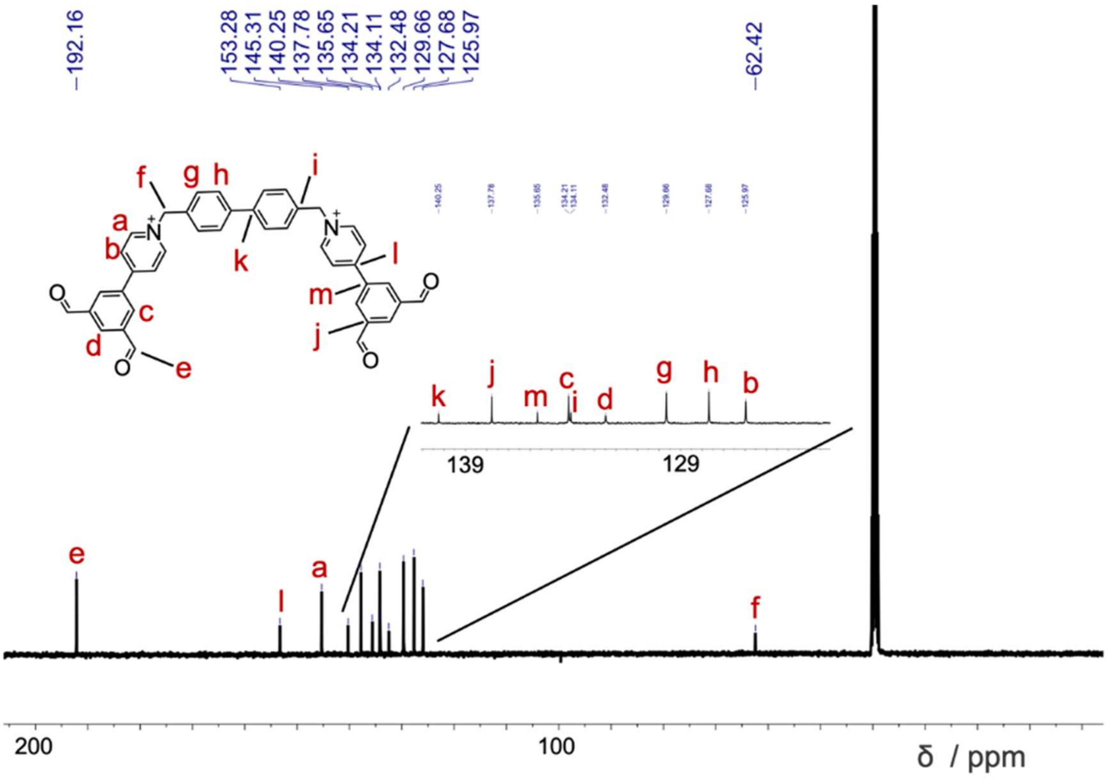

Supplementary Figure 3. NMR characterization. ^{13}C NMR spectrum (100 MHz, CD_3SOCD_3, 298 K) of $\mathbf{1}^{2+}\cdot 2Br^-$.

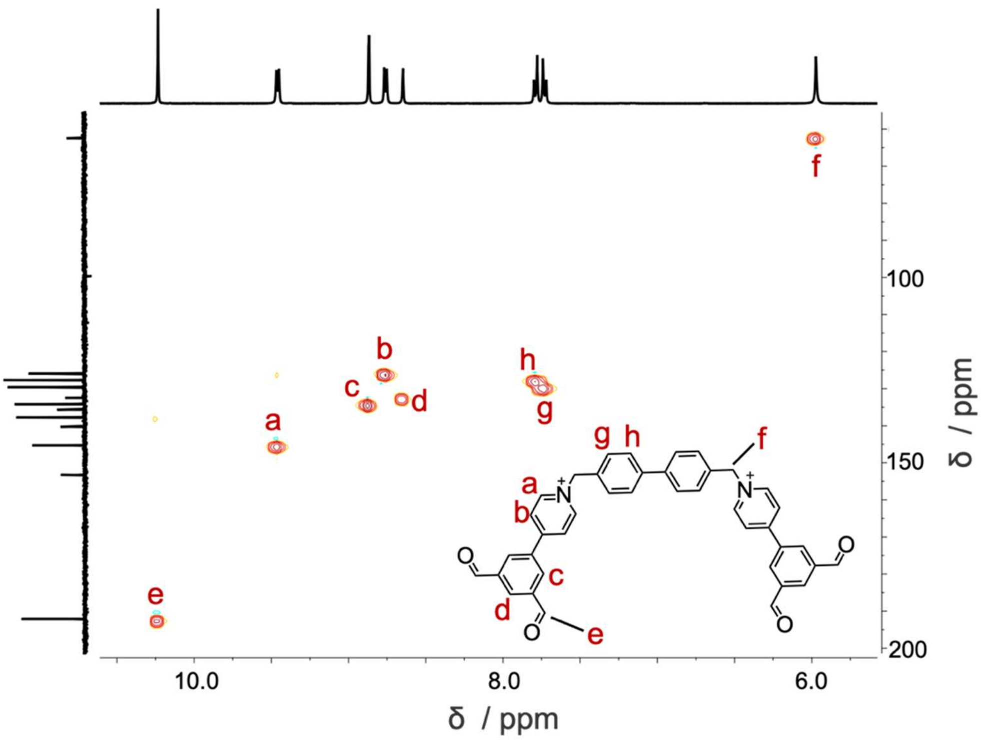

Supplementary Figure 4. **NMR characterization.** HSQC spectrum (CD_3SOCD_3, 298 K) of $\mathbf{1}^{2+}\cdot 2Br^{-}$.

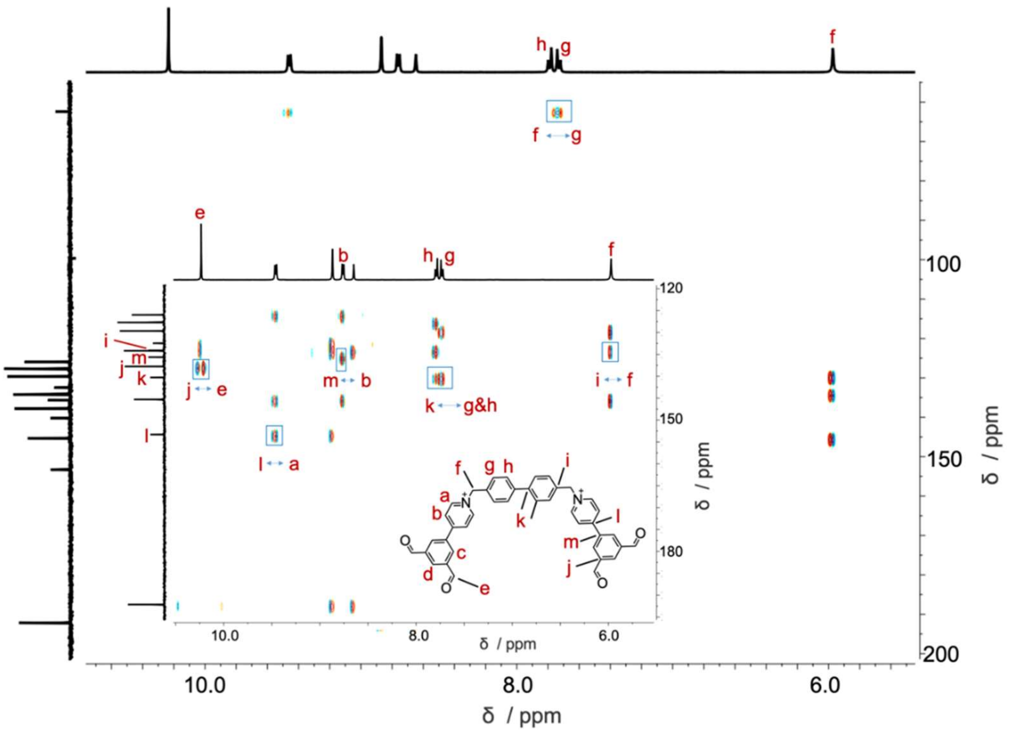

Supplementary Figure 5. NMR characterization. HMBC spectrum (CD_3SOCD_3, 298 K) of $\mathbf{1}^{2+}\cdot 2Br^{-}$. Key correlation peaks are labeled in the spectrum.

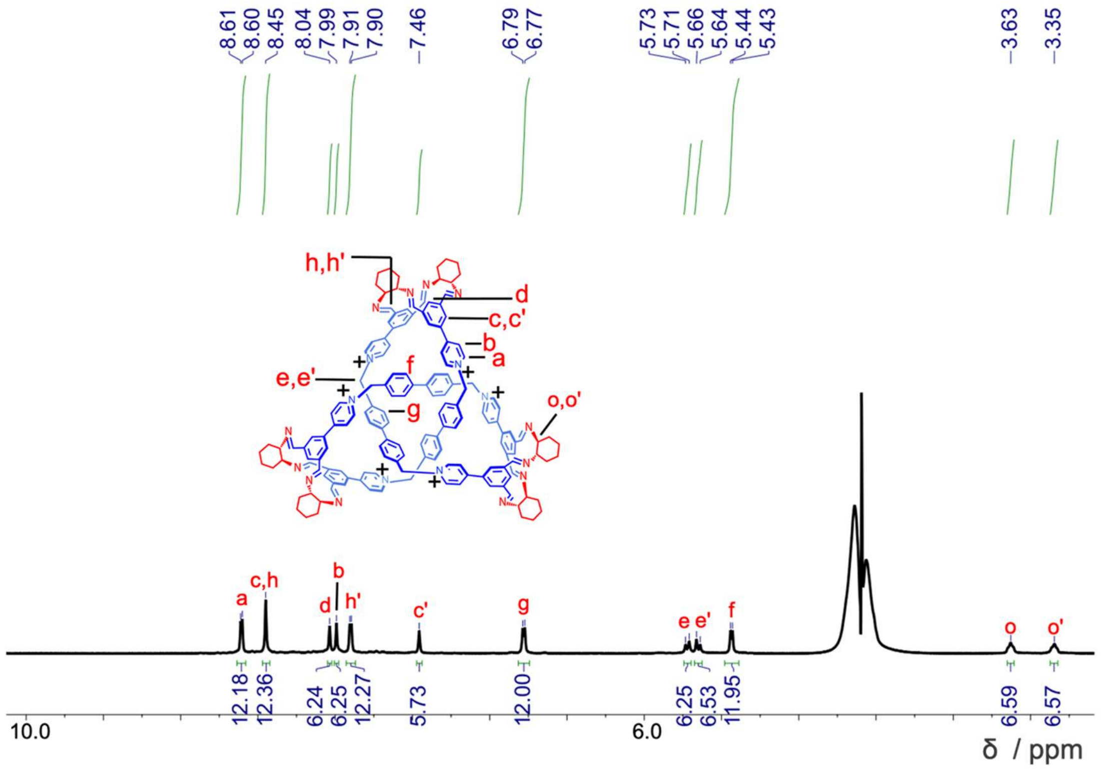

Supplementary Figure 6. NMR characterization. The partial ^{1}H NMR spectrum (600 MHz, H_2O/CD_3SOCD_3 (v/v, 3:1), 298 K) of ***S*-2**$^{6+}\cdot 6Br^{-}$.

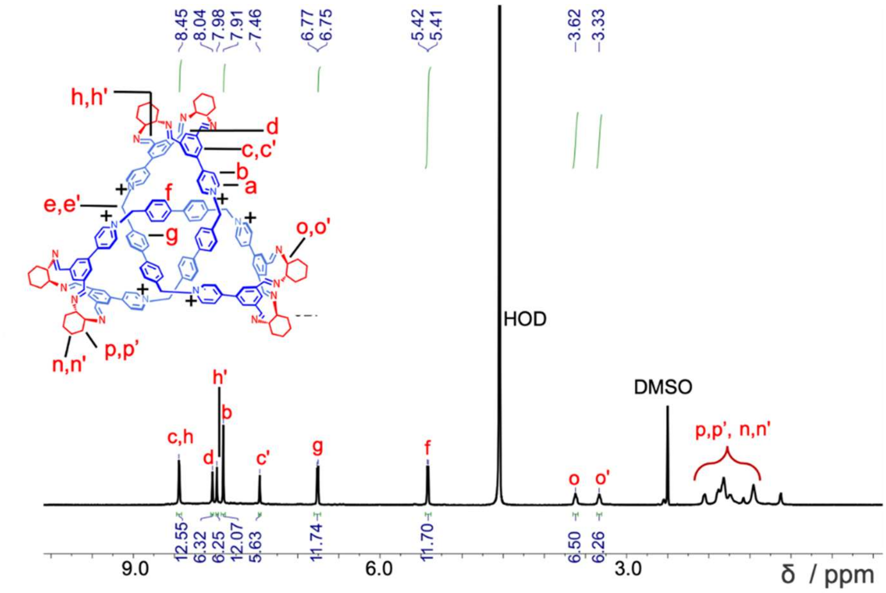

Supplementary Figure 7. NMR characterization. ^{1}H NMR spectrum (600 MHz, D_2O/CD_3SOCD_3 (v/v, 3:1), 298 K) of ***S*-2**$^{6+}\cdot 6Br^{-}$. Different from the spectrum shown in Supplementary Figure 6, a few resonances corresponding to protons *a* and *e*/*e'* disappeared, on account of deuteration.

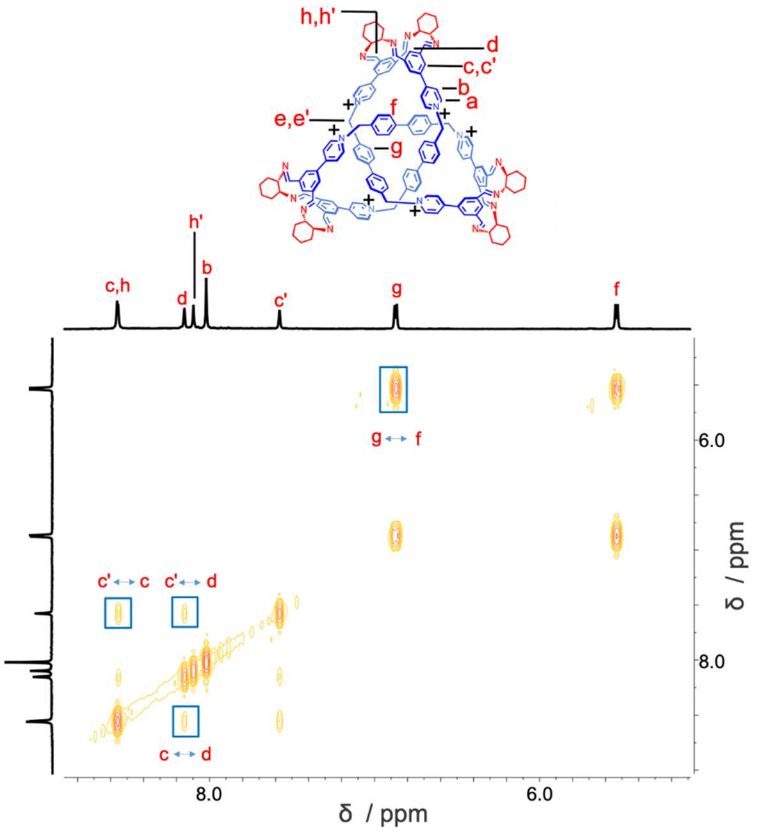

Supplementary Figure 8. NMR characterization. ^{1}H-^{1}H COSY spectrum (600 MHz, D_2O/CD_3SOCD_3 (v/v, 3:1), 298 K) of ***S*-2**$^{6+}\cdot 6Br^{-}$.

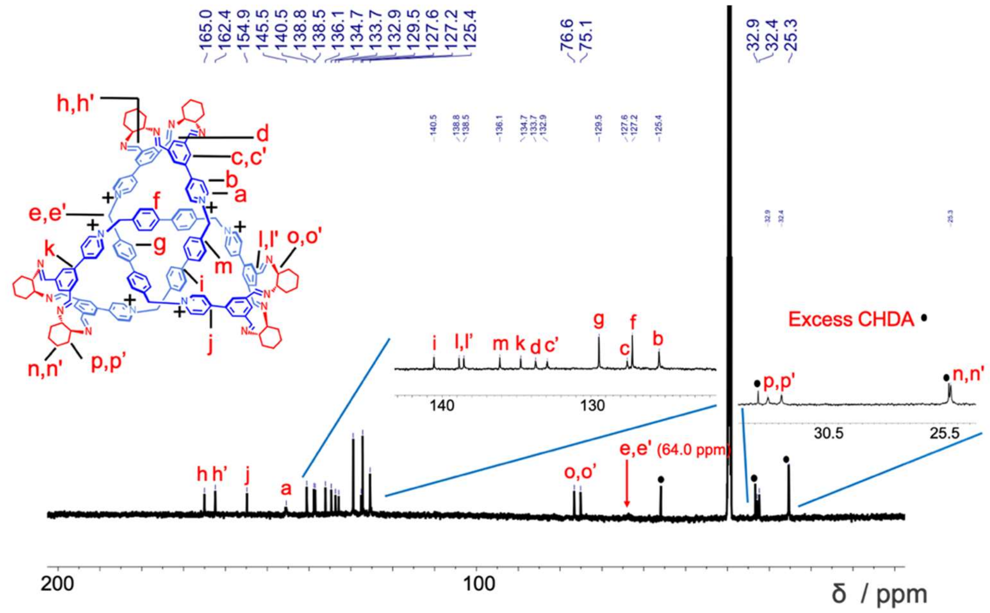

Supplementary Figure 9. NMR characterization. ^{13}C NMR spectrum (150 MHz, D_2O/CD_3SOCD_3 (v/v, 3:1), 298 K) of ***S*-2**$^{6+}\cdot 6Br^{-}$.

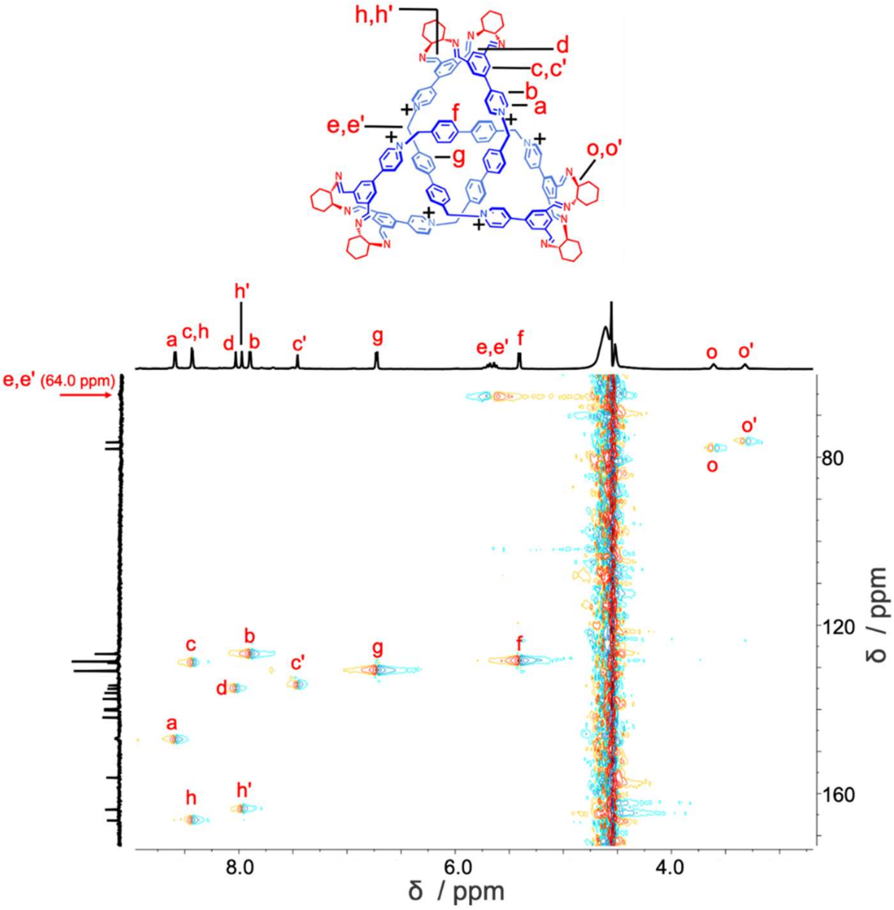

Supplementary Figure 10. NMR characterization. HSQC spectrum (H_2O/CD_3SOCD_3 (v/v, 3:1), 298 K) of ***S*-2**$^{6+}$·6Br^{-}. Key correlation peaks are labeled in the spectrum.

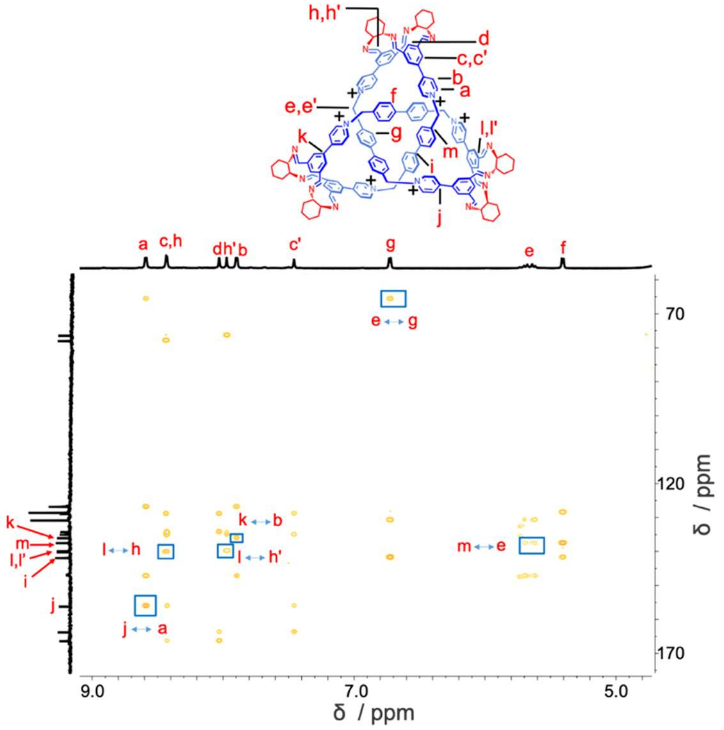

Supplementary Figure 11. NMR characterization. HMBC spectrum (H_2O/CD_3SOCD_3 (v/v, 3:1), 298 K) of ***S*-2**$^{6+}$·$6Br^-$. Key correlation peaks are labeled in the spectrum.

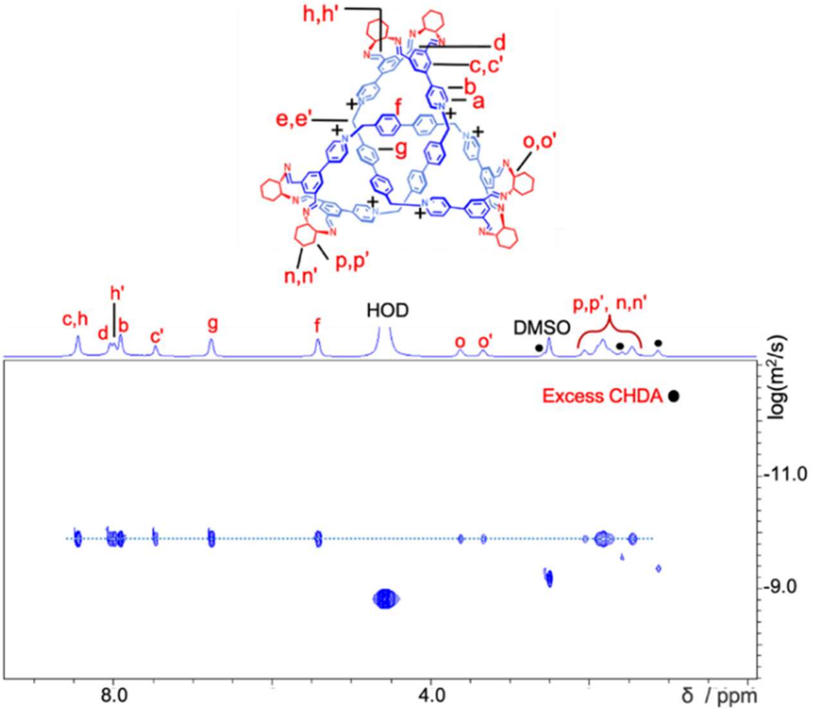

Supplementary Figure 12. NMR characterization. DOSY spectrum of ***S*-2**$^{6+}$·$6Br^-$ in D_2O/CD_3SOCD_3 (v/v, 3:1) at 298 K. The diffusion coefficient (*D*) is about 1.51×10^{-10} m^2/s; The *D* of ***S*-2**$^{6+}$·$6COO^-$ in D_2O is determined to be 1.78×10^{-10} m^2/s.

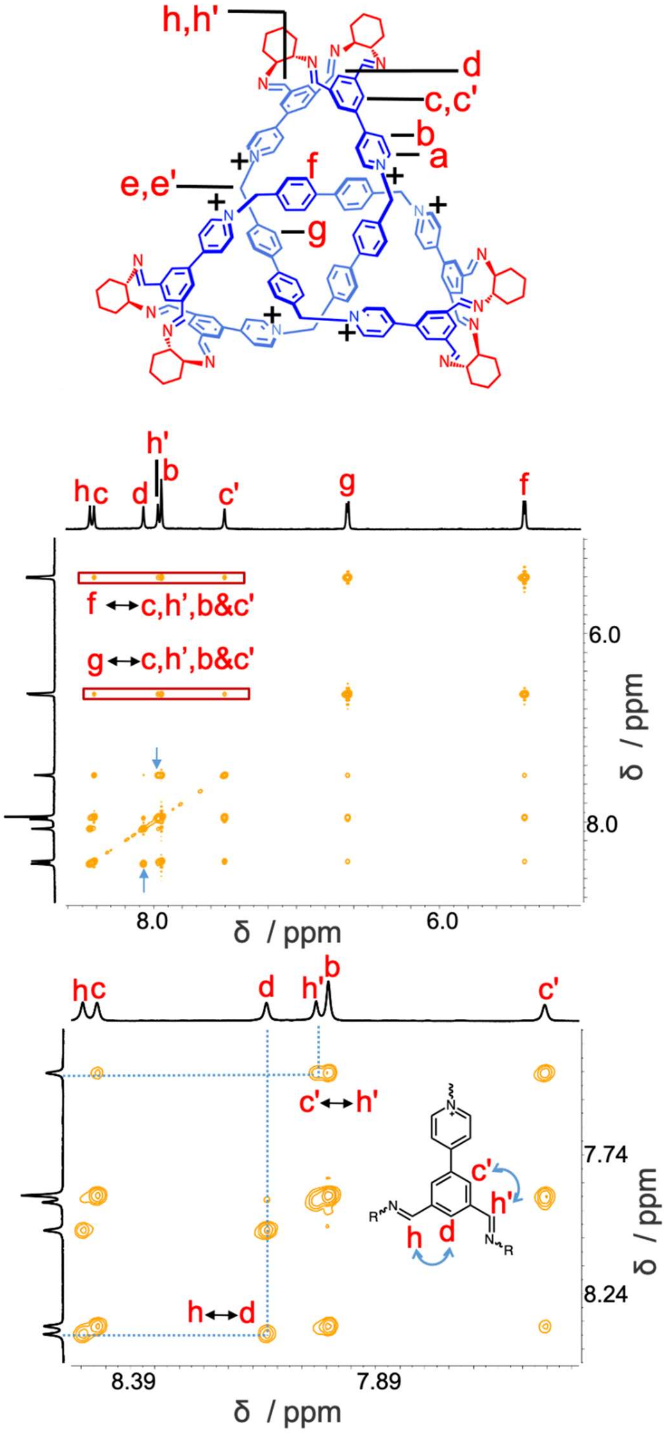

Supplementary Figure 13. NMR characterization. NOESY spectrum of ***S*-2**$^{6+}$·6Br^{-} in D_2O/CD_3SOCD_3 (v/v, 1:1) recorded at 298 K. Key correlation peaks are labeled in the spectrum. Here, higher DMSO content (i.e., 1:1) was used, instead of D_2O/CD_3SOCD_3 (v/v, 3:1) used before. This is because in D_2O/CD_3SOCD_3 (v/v, 3:1), the resonances corresponding to *h* and *c* underwent overlapping. In D_2O/CD_3SOCD_3 (v/v, 1:1), these two resonances slightly shifted, so that the coupling peaks were more clearly observed.

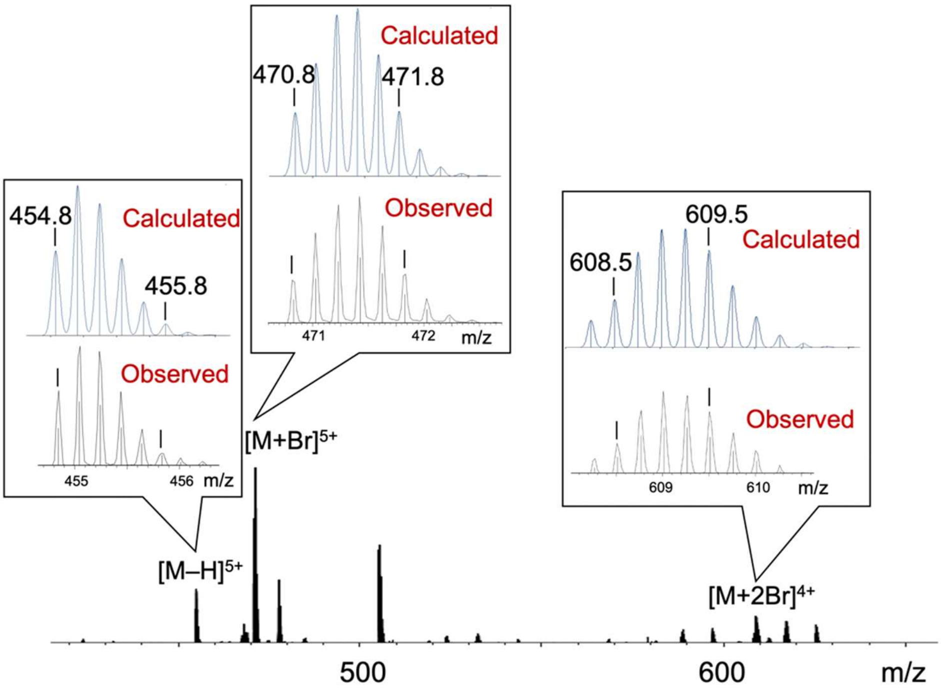

Supplementary Figure 14. ESI-HRMS of *S*-2^{6+}·6Br^{-}. The signals labeled in the spectrum correspond to molecular cations that contain five, five and four charges, respectively, by either gaining Br^{-} counterions or losing a proton.

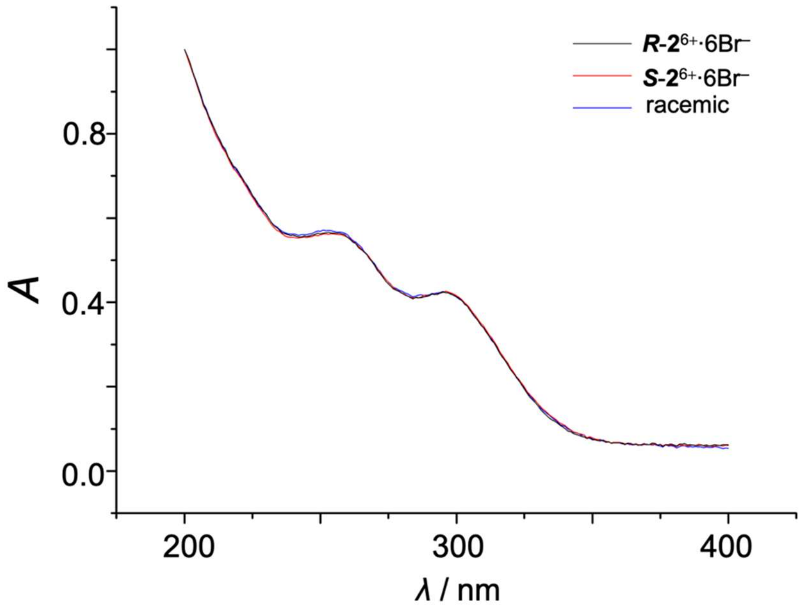

Supplementary Figure 15. UV-Vis absorption spectra of trefoil knots including two enantiomers and the racemic mixture. Normalized partial UV-Vis absorption spectra of the product ***S*-2**$^{6+}$·6Br^{-}, ***R*-2**$^{6+}$·6Br^{-} and their racemic mixture recorded in water at 298 K. The racemic cage mixture was self-assembled by using racemic CHDA. Their UV-Vis absorption spectra are almost identical.

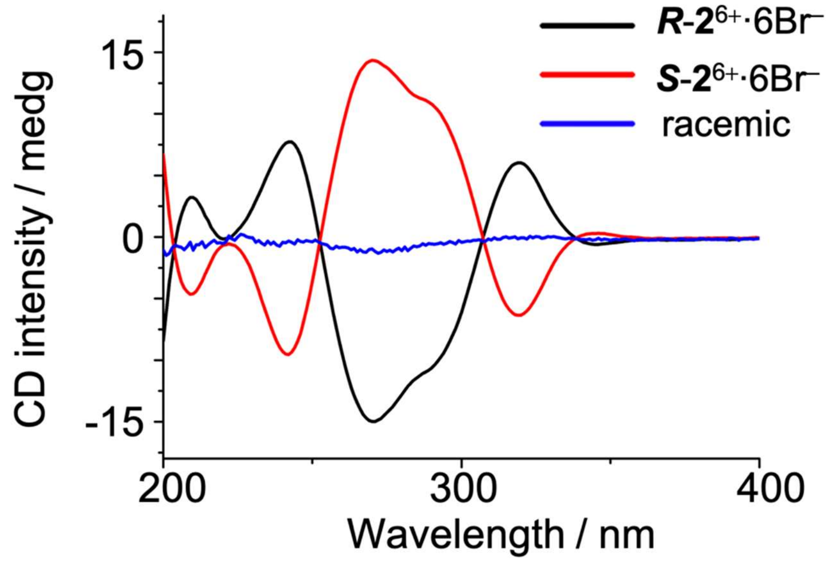

Supplementary Figure 16. CD spectra of trefoil knots including the two enantiomers and their racemic mixture. CD spectra of the product ***S*-2**$^{6+}$·6Br^{-}, ***R*-2**$^{6+}$·6Br^{-} and their racemic mixture (0.04 mM) recorded in water. The racemic trefoil knot mixture was self-assembled by condensing a racemic mixture of CHDA and **1**$^{2+}$·2Br^{-} in water. Once self-assembled, the trefoil knot, including both enantiomers and racemic mixture, was observed remarkably inert within hours, after dilution of the solution to 0.04 mM.

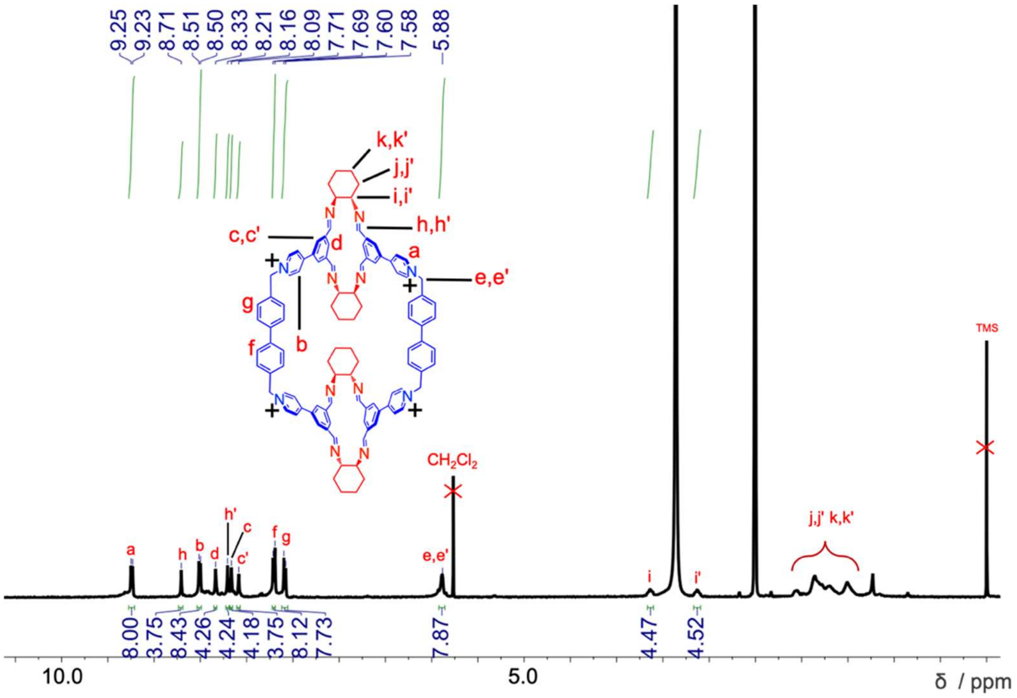

Supplementary Figure 17. NMR characterization. The partial ^{1}H NMR spectrum (600 MHz, CD_3SOCD_3, 298 K) of ***S*-3**$^{4+}$·4Br^{-}.

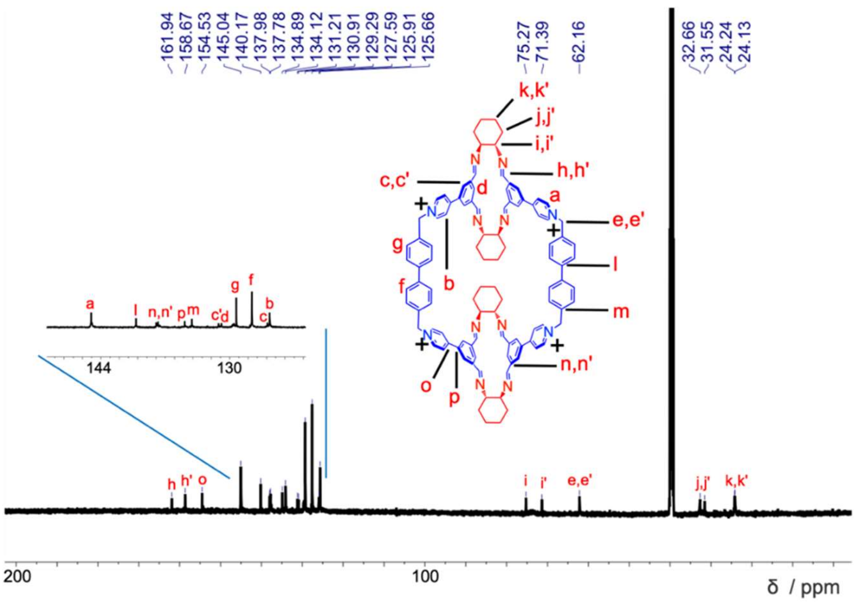

Supplementary Figure 18. NMR characterization. ^{13}C NMR spectrum (150 MHz, CD_3SOCD_3, 298 K) of ***S*-3**$^{4+}$·4Br^{-}.

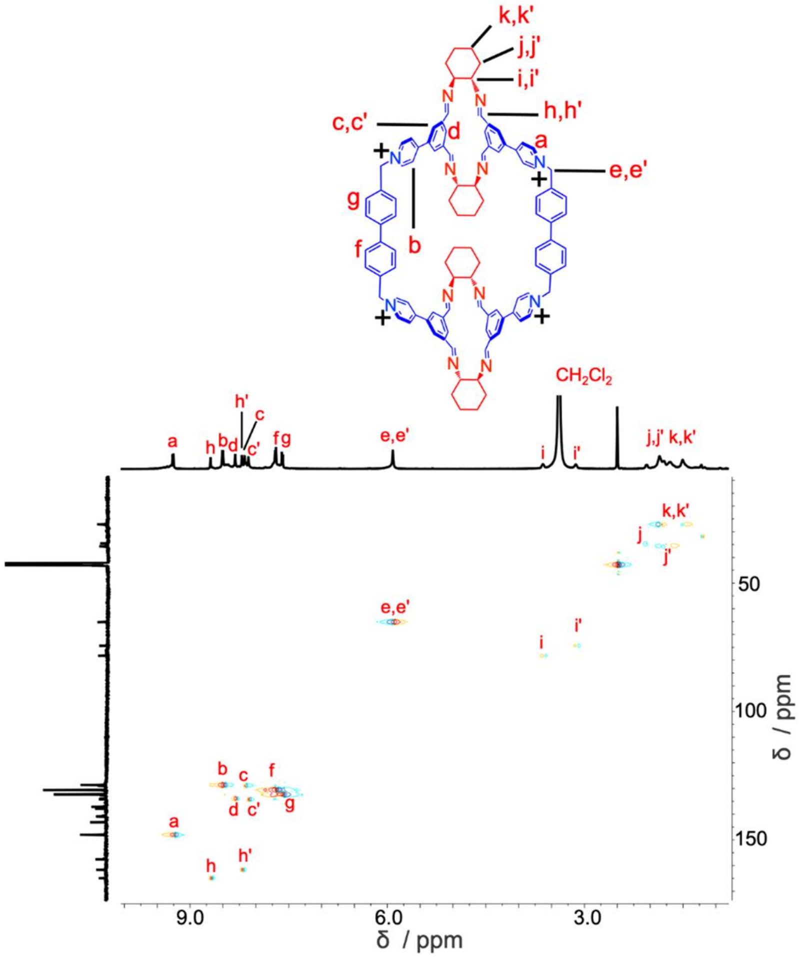

Supplementary Figure 19. NMR characterization. HSQC spectrum (CD_3SOCD_3, 298 K) of ***S*-3**$^{4+}$·4Br^{-}. Key correlation peaks are labeled in the spectrum.

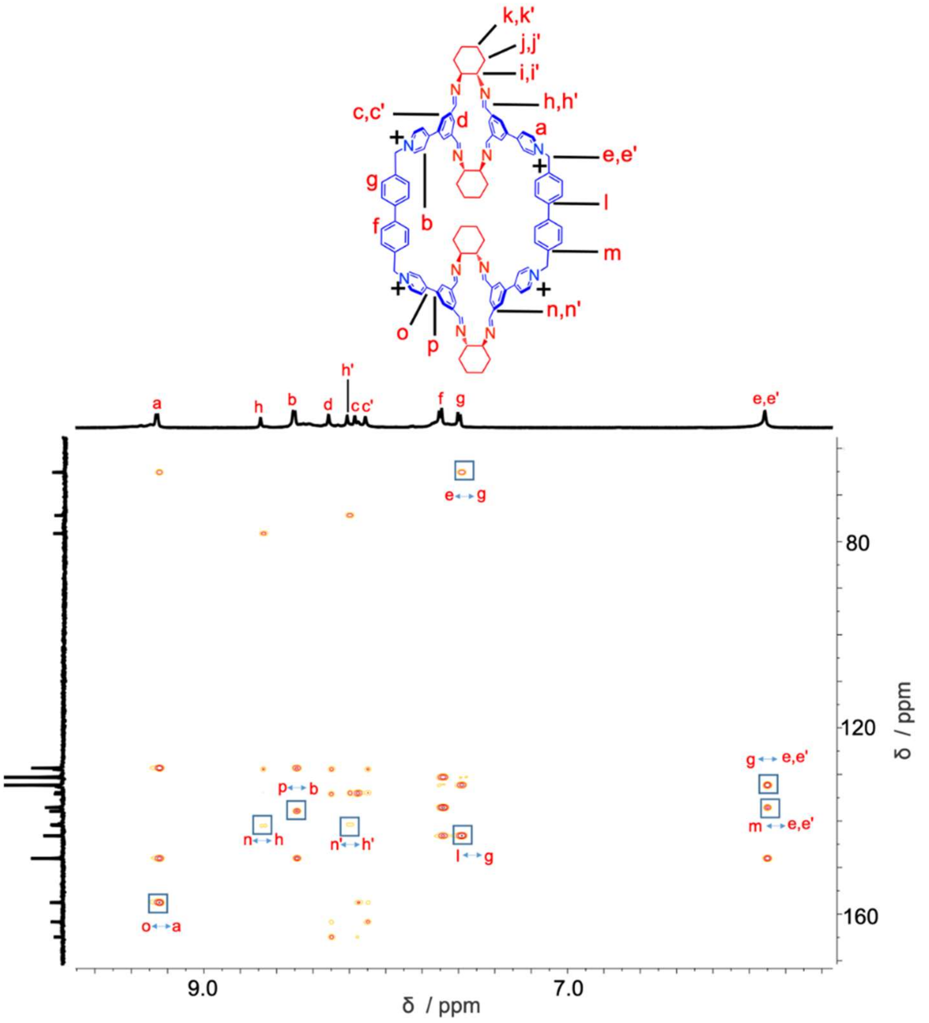

Supplementary Figure 20. NMR characterization. HMBC spectrum (CD_3SOCD_3, 298 K) of ***S*-3**$^{4+}$·$4Br^{-}$. Key correlation peaks are labeled in the spectrum.

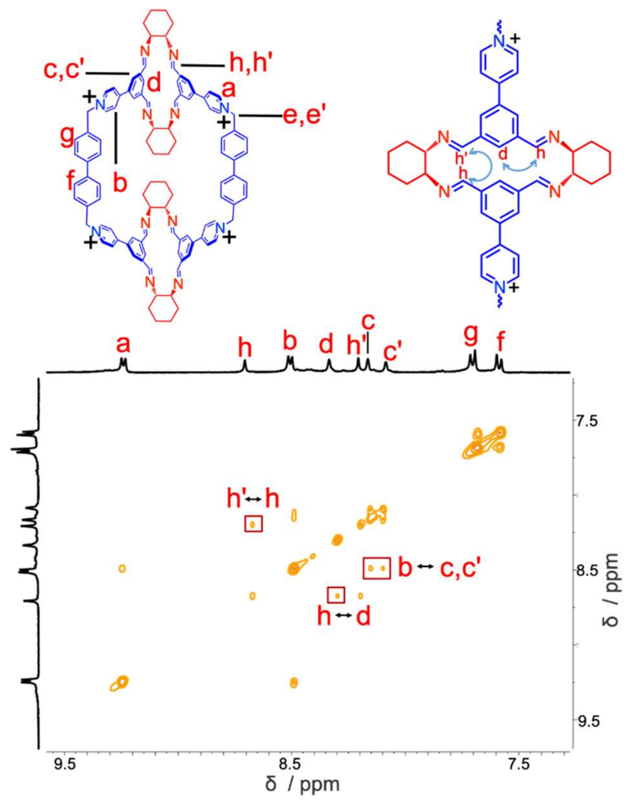

Supplementary Figure 21. NMR characterization. NOESY spectrum (CD_3SOCD_3, 298 K) of ***S*-3**$^{4+}$·4Br^{-}. Key correlation peaks are labeled in the spectrum.

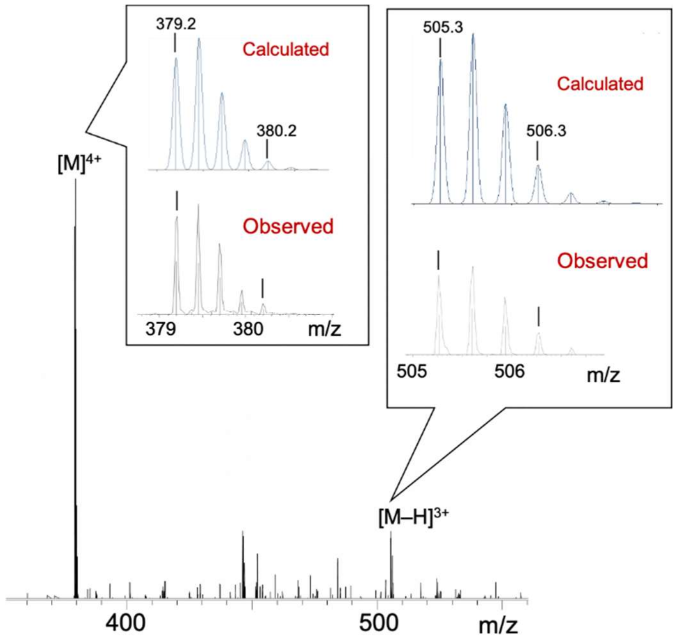

Supplementary Figure 22. ESI-HRMS of *S*-3^{4+}·4Br^{-}. The signals labeled in the spectrum correspond to molecular cations that contain four and three charges, respectively.

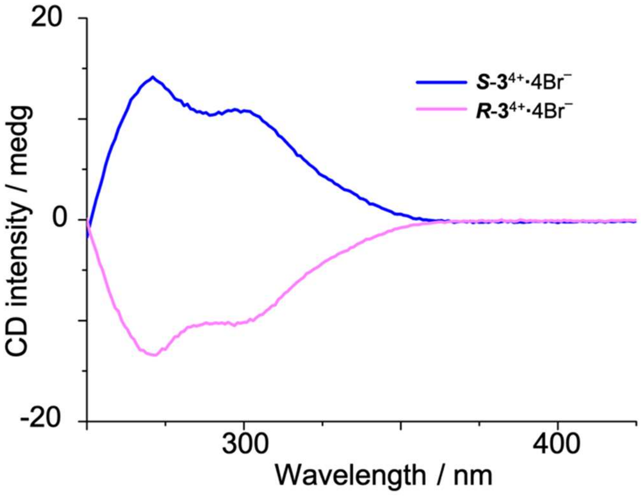

Supplementary Figure 23. CD spectra of macrocycles including the two enantiomers. CD spectra of the product ***S*-3**$^{4+}$·4Br^{-} and ***R*-3**$^{4+}$·4Br^{-} (0.06 mM) recorded in DMSO.

4. Investigations of the self-assembly process

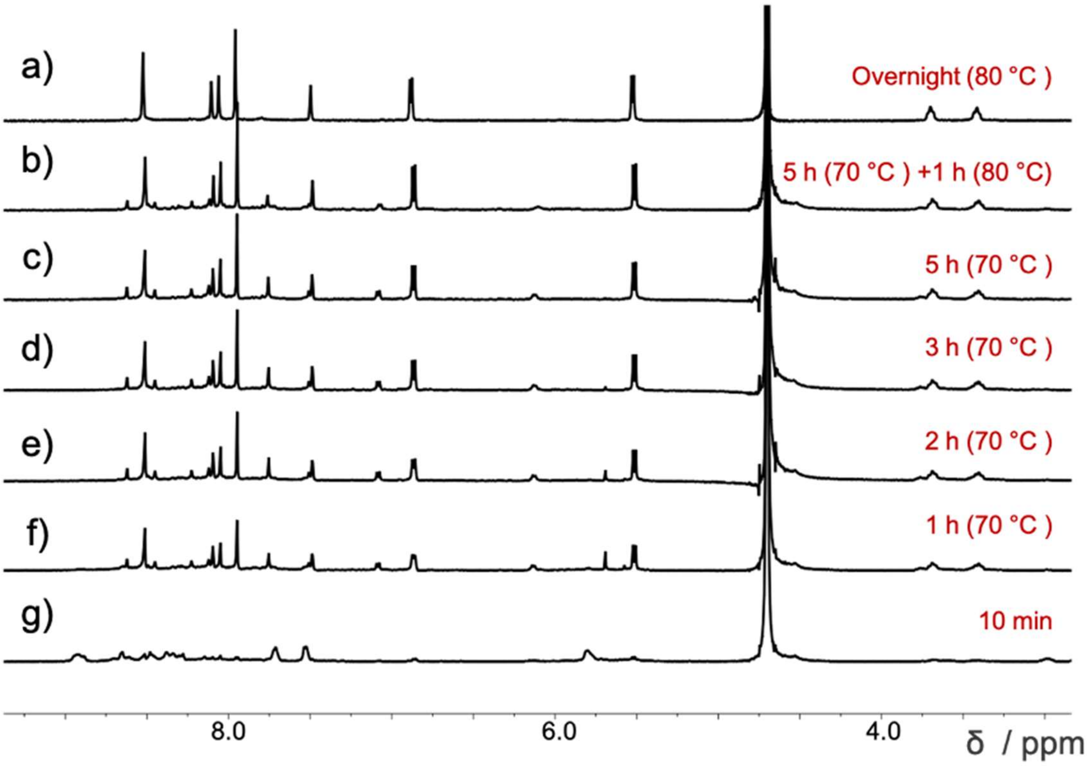

Supplementary Figure 24. The self-assembly process of *S*-2^{6+}·6Br^{-}. The partial ^{1}H NMR spectra (500 MHz, D_2O, 298 K) of 1:2 mixture of **1**$^{2+}$·2Br^{-} (3 mM) and (*SS*)-CHDA recorded after heating the mixture for different amount of time.

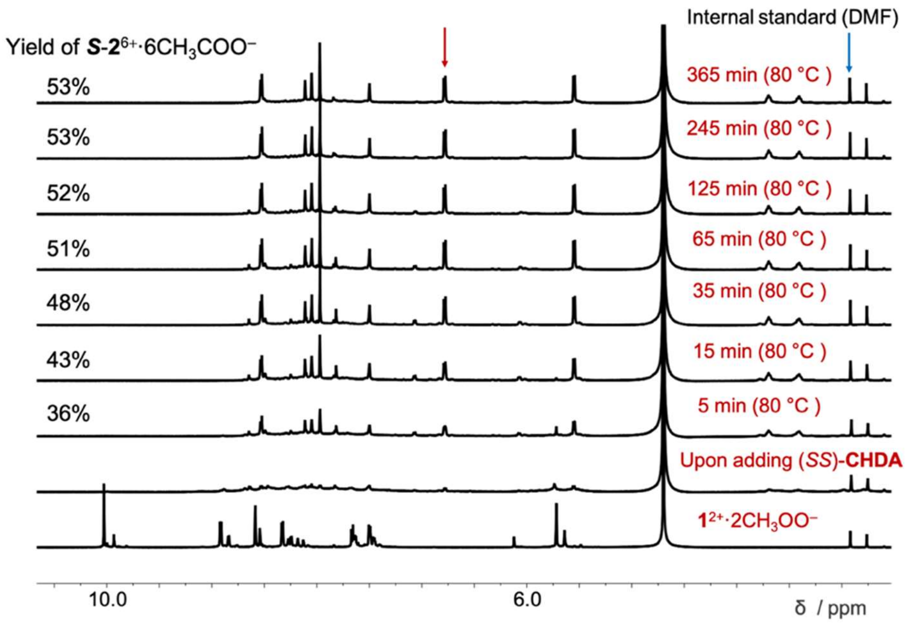

Supplementary Figure 25. **The self-assembly process of *S*-2^{6+}·$6CH_3COO^{-}$.** The partial ^{1}H NMR spectra (600 MHz, D_2O, 298 K) of 1:2 mixture of **1**$^{2+}$·$2CH_3COO^{-}$ (7 mM) and (*SS*)-CHDA recorded after heating the mixture for special amount of time, yielding the predominant product ***S*-2**$^{6+}$·$6CH_3COO^{-}$. DMF was added as an internal standard to calculate the yield of ***S*-2**$^{6+}$·$6CH_3COO^{-}$ at each time, so that the self-assembly kinetics could be evaluated. However, we discovered that ***S*-2**$^{6+}$·$6CH_3COO^{-}$ would undergo decomposition partially even in room temperature due to the basicity of CH_3COO^{-}, which means that the yields would be more than what we calculated.

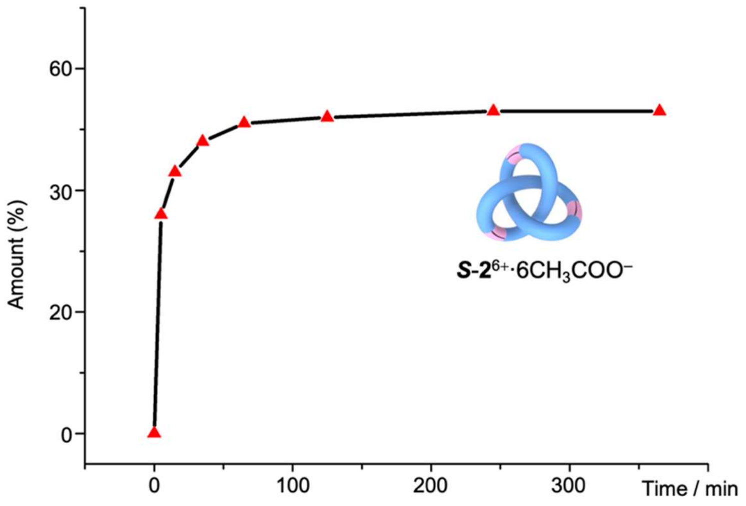

Supplementary Figure 26. The kinetic formation curve of 2^{6+}·$6CH_3COO^{-}$. A plot of the yields of ***S*-2**$^{6+}$·$6CH_3COO^{-}$ versus reaction time. The yields of the trefoil knot at each time were measured by using the ^{1}H NMR spectroscopic results in Supplementary Figure 25).

The studies of the self-assembly kinetics were performed by recording the ^1H NMR spectra (Supplementary Figure 24) at different time on the reaction course. $\mathbf{1}^{2+}\cdot 2CH_3COO^-$, a more water-soluble counterpart of $\mathbf{1}^{2+}\cdot 2Br^-$, was prepared via couterion exchange. We mixed $\mathbf{1}^{2+}\cdot 2CH_3COO^-$ and (*SS*)-CHDA in D_2O at 80 ºC. The ^1H NMR spectra of this mixture were recorded (Supplementary Figure 25) during the self-assembly process. At the early stage of the reaction course, ***S***-$\mathbf{2}^{6+}$ was observed, which was companied with various intractable kinetic byproducts. The byproducts gradually disappeared, leaving ***S***-$\mathbf{2}^{6+}$ as the predominant product in the ^1H NMR spectrum. A few acidic protons in the trefoil knot underwent deuteration and their resonances disappeared. The successful self-assembly of ***S***-$\mathbf{2}^{6+}\cdot 6CH_3COO^-$ also indicates that Br^- is not an indispensable template. By integrating one of the resonances corresponding to the trefoil knot (marked with red arrow in Supplementary Figure 25), the relative concentrations of the trefoil knot at each time were successfully calculated. A plot of the yields versus time was obtained (see Supplementary Figure 26), showing that the self-assembly of this trefoil knot was almost complete within 100 min at 80 ºC. However, we discovered that ***S***-$\mathbf{2}^{6+}\cdot 6CH_3COO^-$ would undergo decomposition partially even at room temperature due to the basicity of CH_3COO^-, which means that the yields would be more than what we calculated.

Another question is that, what is the kinetic byproducts during the self-assembly? We thus performed the self-assembly of the trefoil knot at room temperature, in order to increase the life-time of the byproducts. Here, H_2O/CD_3SOCD_3 (3:1, v/v) was used to avoid deuteration. Due to the instability of this intermediate, we were not able to isolate this compound and characterize it in a more conclusive manner. Mass spectrum (Supplementary Figure 27) clearly indicated the byproduct is a [4+8] compound, namely that it is composed of four equivalents of the tetraaldehyde and eight equivalents of the bisamine. The DOSY spectrum (Supplementary Figure 28c) clearly indicates that this [4+8] byproduct has a slightly smaller diffusion coefficient compared to the trefoil knot, implying that this byproduct has a slightly larger size or molecular weight compared to the trefoil knot ([3+6] product).

^{1}H NMR (Supplementary Figure 28b) spectra indicate that this [4+8] byproduct ***S*-X**$^{8+}$·8Br$^-$ is a Solomon link. In the ^{1}H NMR spectrum, some of the resonances in this [4+8] byproduct also underwent remarkable upfield shift as occurred in the case of trefoil knot, indicating this [4+8] byproduct also has an intertwined architecture. Of course, it is also possible that the [4+8] byproduct is a [2]catenane composed of two [2+4] macrocycles. However, this [2+4] macrocycle contains two bulky "speed bumps". As a consequence, the ring-ring rotaxane motion should be relatively slow, which leads to two sets of ^{1}H NMR resonances, including the protons inside and outside. This hypothesis is not consistent with the ^{1}H NMR spectrum in which only one set of resonances were observed.

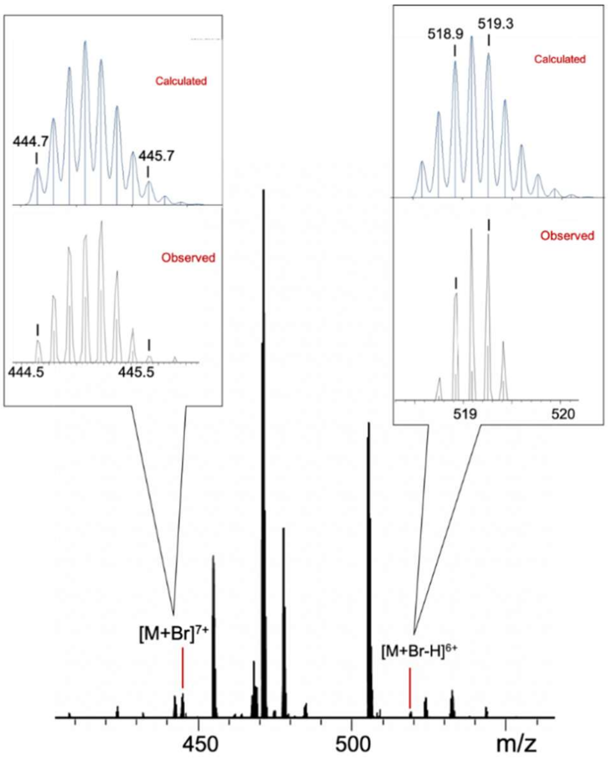

Supplementary Figure 27. ESI-HRMS of a mixture of *S*-2^{6+}·6Br$^-$ and *S*-X^{8+}·8Br$^-$. The signals labeled in the spectrum correspond to molecular cations that contain seven, and six charges, respectively, which belongs to a [4+8] product ***S*-X**$^{8+}$·8Br$^-$.

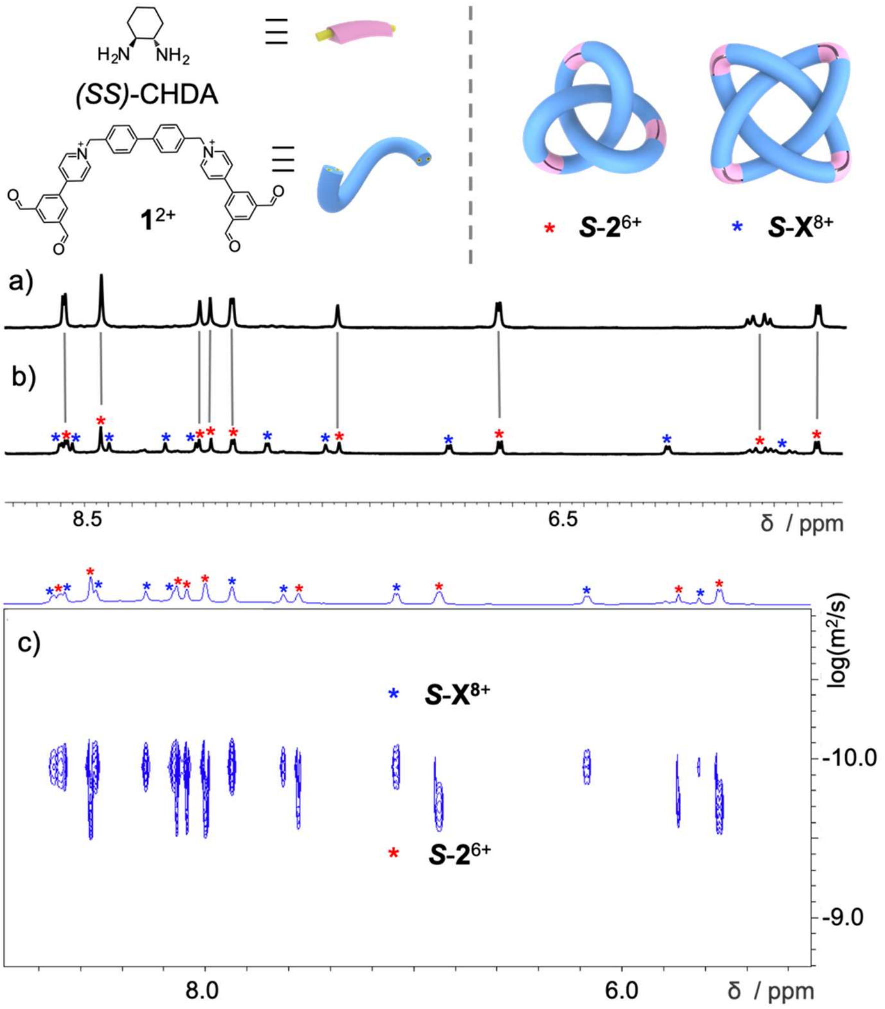

Supplementary Figure 28. **The self-assembly products at room temperature.** The partial ^{1}H NMR spectra (500 MHz, 298 K) of (a) the trefoil knot ***S*-2**$^{6+}$·6Br$^{–}$ in H_2O/CD_3SOCD_3 (v/v, 3:1) and (b) a 1:2 mixture of **1**$^{2+}$·2Br$^{–}$ (2 mM) and (*SS*)-CHDA (4 mM) in H_2O/CD_3SOCD_3 (v/v, 3:1) recorded after it was placed at room temperature for 12 h. Here, H_2O was used instead of D_2O in order to avoid deuteration. (c) DOSY spectrum of the sample prepared in the same procedure as that in (b), using D_2O instead of H_2O at 298 K.

The chirality of the macrocycle ***S*-3**$^{4+}$·4Br$^{–}$ renders a few of its protons including protons *e*, *h* and *c* diastereotopic (Supplementary Figure 17). It was observed that the ^{1}H NMR of ***S*-3**$^{4+}$·4Br$^{–}$ is temperature dependent. At higher temperature, these resonances corresponding to two diastereotopic protons gradually underwent coalescence, indicating the flipping of the building blocks within the macrocycle (Supplementary Figure 29) on ^{1}H NMR timescale at elevated temperature. The energy

barrier (ΔG) was determined to be about 64.0 kJmol^{-1}, by using Eyring equation (Supplementary Figure 29). While in the case of ***S*-2**$^{6+}$·6Br^{-}, the peaks barely changed at 60 °C (Supplementary Figure 30), which demonstrated that intertwined nature of ***S*-2**$^{6+}$·6Br^{-} prevented intramolecular flipping.

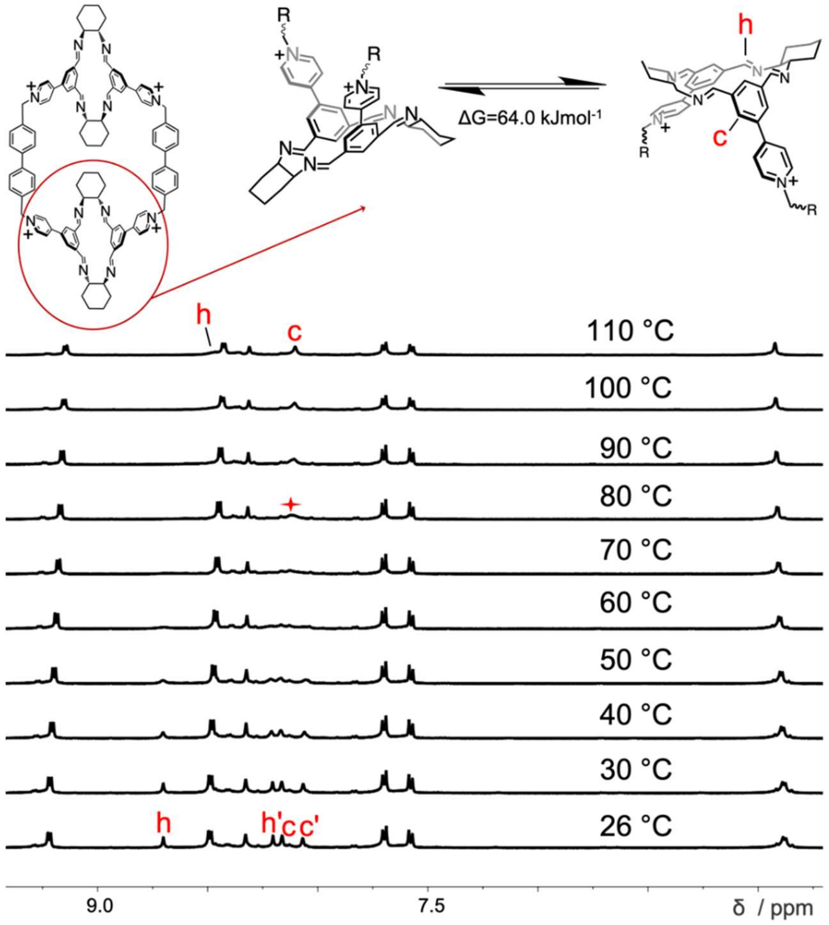

Supplementary Figure 29. Calculation of the energy barrier of the macrocycle undergoing intramolecular flipping. The partial ^{1}H NMR spectra (500 MHz, CD_3SOCD_3) of ***S*-3**$^{4+}$·4Br^{-} recorded at different temperatures.

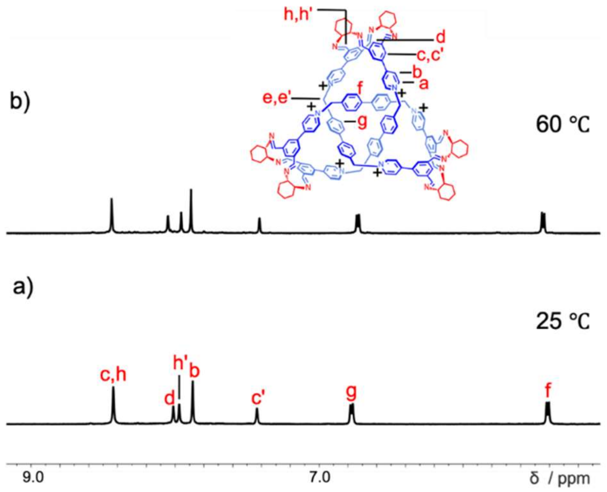

Supplementary Figure 30. Temperature-independent properties of *S*-2^{6+}·6Br^{-}. The partial ^{1}H NMR spectra (500 MHz, D_2O/CD_3SOCD_3 (3:1, v/v)) of ***S*-2**$^{6+}$·6Br^{-} recorded at a) 25 °C and b) 60 °C, respectively. The ^{1}H NMR spectra seemed to be temperature independent.

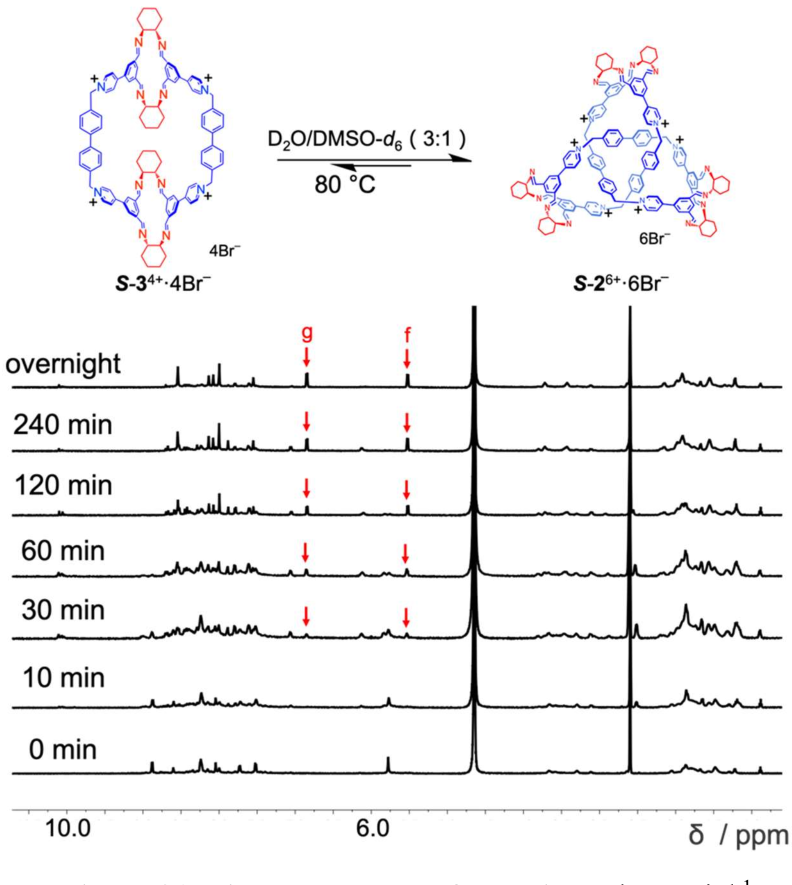

Supplementary Figure 31. Ring to knot transformation. The partial ^{1}H NMR spectra (500 MHz, D_2O/CD_3SOCD_3 (3:1), 298 K) of ***S*-3**$^{4+}$·4Br^{-} recorded after being heated at 80 °C for special amount of time. ***S*-3**$^{4+}$·4Br^{-} transferred gradually to the knot ***S*-2**$^{6+}$·6Br^{-}, which was driven by hydrophobic effect in water.

Upon dissolved in D_2O/CD_3SOCD_3 (3:1), the ^{1}H NMR spectra (Supplementary Figure 31) of ***S*-3**$^{4+}\cdot$4Br$^-$ were recorded. Heating the solution of ***S*-3**$^{4+}\cdot$4Br$^-$ for 16 h at 80 °C transferred the ring to the knot ***S*-2**$^{6+}\cdot$6Br$^-$.

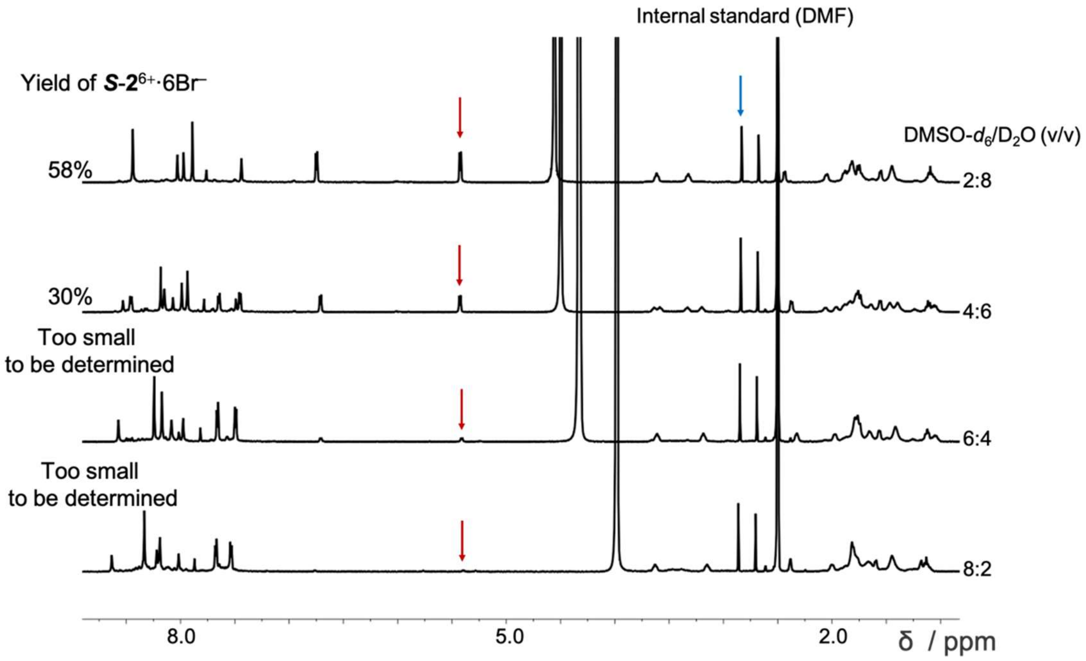

Supplementary Figure 32. Solvent effect on the formation of *S*-2$^{6+}\cdot$6Br$^-$. ^{1}H NMR spectra (500 MHz, 298 K) of 1:2 mixture of **1**$^{2+}\cdot$2Br$^-$ (2 mM) and (*SS*)-CHDA (4 mM) recorded after heating the mixture at 80 °C for 12 h in the mixtures of CD_3SOCD_3 and D_2O with different ratios. DMF was added as an internal standard to calculate the yields of the knot. By comparing the integrations of the resonances of DMF (blue arrow) and one proton (red arrow) in the trefoil knot, the yields of the latter were determined.

We further investigated the impact of the D_2O/CD_3SOCD_3 ratio on the yields of the trefoil knot ***S*-2**$^{6+}$. We used D_2O/CD_3SOCD_3 mixtures to do the measurement, whose ratios were 2:8, 4:6, 6:4, and 8:2 (Supplementary Figure 32). An internal standard namely DMF was added into the samples to help the calculation of the yields of the trefoil knot ***S*-2**$^{6+}\cdot$6Br$^-$ in different solvent systems. After the self-assembly systems was heated at 80 °C for 12 h and reached the equilibria, ^{1}H NMR spectra were recorded (Supplementary Figure 32). With the increasing ratio of D_2O/CD_3SOCD_3, the yield of ***S*-2**$^{6+}\cdot$6Br$^-$ increased from very low (i.e., which is too low to be precisely determined by integrating the resonances) to 58%. Such experiment indicates the important role of

hydrophobic effects in water. The yield of ***S***-**2**$^{6+}$·6Br^{-} self-assembled in pure water is difficult to calculate accurately due to the relatively poor water solubility of the tetraformyl precursor **1**$^{2+}$·2Br^{-} and trefoil knot ***S***-**2**$^{6+}$·6Br^{-}.

5. Theoretical calculations

The four right-handed trefoil knots in the main text were optimized by using the density functional theory (DFT) at the BP86-D3/6-311G(d) level with the Gaussian 16 package[2]. The solvent effect of water was included with the polarizable continuum model (PCM) using solvent accessible surface.

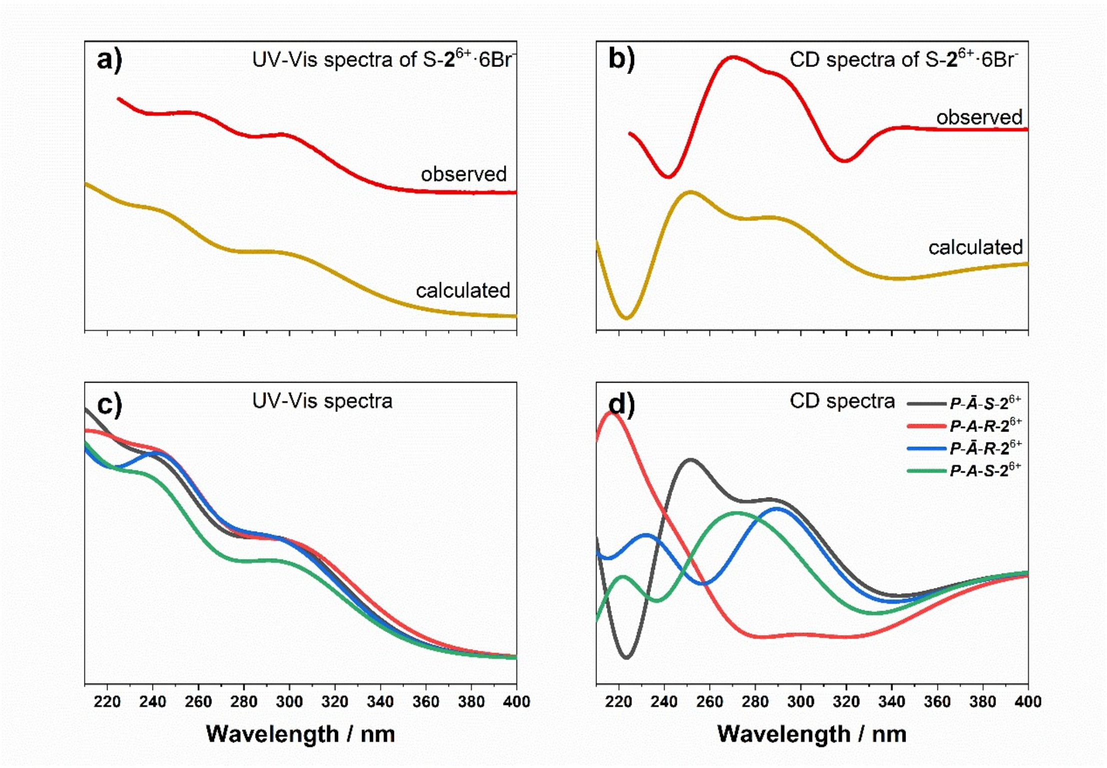

Supplementary Figure 33. Theoretical calculation. Comparison of the experimental (red traces) and theoretical (yellow traces) spectra including (a) UV-Vis and (b) CD spectra. The theoretical spectra were calculated based on the structure of ***P-Ā-S*-2**$^{6+}$ which was also predicted by using DFT. The experimental spectra were obtained by using sample ***S*-2**$^{6+}$·6Br^{-}. The experimental ones are well-consistent with the calculated ones. The comparison of theoretical (c) UV-Vis and (d) CD spectra based on the predicted structures, namely ***P-Ā-S***-2^{6+}, ***P-A-R***-2^{6+}, ***P-Ā-R***-2^{6+} and ***P-A-S***-2^{6+}, respectively.

The UV-Vis and electronic circular dichroism (CD) spectra for the optimized geometries were predicted by the time-dependent DFT (TD-DFT) at the wB97XD/6-311G(d) level with 500 states by the Gaussian 16 package. The solvent effect was also considered. Both the UV-Vis and CD spectra were broadened using Gaussian functions by Multiwfn[3]. The full width at half maximum (FWHM) was set as 0.8 eV.

The radius obtained from the molecular model is defined by the distance between the center point of the knot and one of the marginal atoms, which is 13.2 Å. Therefore, the diameter calculated from the molecular model is about 26.4 Å, which is consistent with our experimental result (26 Å).

Supplementary References

1 Greenaway, R. L. et. al. High-throughput discovery of organic cages and catenanes using computational screening fused with robotic synthesis. *Nat. Commun.* **9**, 2849 (2018).

2 Frisch, M. et al. Gaussian 16 Revision B. 01. 2016. (Gaussian Inc., Wallingford CT).

3 Lu, T. & Chen, F. Multiwfn: A multifunctional wavefunction analyzer, *J. Comput. Chem.* **33**, 580–592 (2012).
