# Supplementary material for: A trefoil knot self-templated through imination in water
Source: Nat Commun. 2022 Jun 21;13:3557. doi: 10.1038/s41467-022-31289-1 (PMC9213439; doi:10.1038/s41467-022-31289-1)
Supplement: Supplementary file 3 — Description of Additional Supplementary Files [file 41467_2022_31289_MOESM3_ESM.pdf]

Description of Additional Supplementary Files

File Name: Supplementary Data 1

Description: Cartesian Coordinates and Energies of the Four Diastereoisomers
