# Supplementary material for: A trefoil knot self-templated through imination in water
Source: Nat Commun. 2022 Jun 21;13:3557. doi: 10.1038/s41467-022-31289-1 (PMC9213439; doi:10.1038/s41467-022-31289-1)
Supplement: Supplementary file 4 — Supplementary Data File 1 [file 41467_2022_31289_MOESM4_ESM.pdf]

Supplementary Data 1

Cartesian Coordinates and Energies of the Four Diastereoisomers

***P*-*Ā*-*S*-2**$^{6+}$ (ΔG = 0 kcal/mol)
Total SCF energy (BP86-D3/6-311G(d)/PCM(water)): -7021.284619 a.u.
Thermal correction to Gibbs free energy at 298.15 K: 2.485123 a.u.
Gibbs free energy at 298.15 K (BP86-D3/6-311G(d)/PCM(water)): -7018.799496 a.u.

C	-6.03307500	0.13064000	-3.41884400
C	-5.71951900	-0.76362100	2.46339700
C	-5.36259000	-0.29655700	3.74831200
C	-4.98598600	1.02298900	3.94098100
N	-4.93605100	1.89693000	2.89904700
C	-5.28017800	1.48191500	1.64596700
C	-5.67974300	0.18063400	1.40857500
C	-5.02402800	-6.07448900	-2.60724300
C	-3.71246300	-5.93554500	-3.08101800
C	-2.63421700	-6.61919800	-2.46424500
C	-2.91252400	-7.43643100	-1.35146100
C	-4.21757600	-7.56131600	-0.84654500
C	-5.27200100	-6.90595500	-1.49563900
C	-6.11904800	-5.27294900	-3.19472100
N	-7.32458500	-5.40440100	-2.78797900
C	-4.46764400	-8.25982300	0.42963200
N	-3.53260600	-8.42248500	1.29271400
C	-3.92960700	-8.97087600	2.58672800
C	-8.34847300	-4.51838600	-3.31848700
C	-5.63800300	-4.54082700	2.64422000
C	-5.37617100	-3.19125200	2.90902800
C	-6.07043600	-2.17170400	2.21954600
C	-7.03306500	-2.52164100	1.25595800
C	-7.32445600	-3.87518900	1.00023800
C	-6.63858600	-4.87212100	1.70962600
C	-4.79290400	-5.58716400	3.25039500
N	-5.01365800	-6.83184000	3.05749700
C	-8.23855000	-4.28468800	-0.08504100
N	-8.51187800	-3.52382200	-1.08153400
C	-9.30394200	-4.12538100	-2.15357100
C	-4.06167600	-7.79284900	3.59485200
C	-4.11986000	-0.14195200	-1.72688300
C	-3.41958800	-0.92193800	-0.80128200
C	-3.84507300	-2.22485200	-0.47318400
C	-5.02149400	-2.70425800	-1.08661400

C	-5.74272200	-1.91715100	-1.98500300
C	-5.28724100	-0.63381100	-2.33458800
C	-3.07719900	-3.06234100	0.47960000
C	-3.02298300	-4.46062100	0.32441200
C	-2.25535500	-5.25731800	1.17941300
C	-1.51509700	-4.66754500	2.21611800
C	-1.60220400	-3.27731100	2.41614200
C	-2.37802700	-2.48853900	1.56519500
C	-0.61811800	-5.50965700	3.09300200
C	-1.23876000	-6.39068000	-2.87982500
C	-0.88033900	-5.40415400	-3.83121600
C	0.44561700	-5.10888300	-4.08956700
N	1.45039100	-5.75991900	-3.43988600
C	1.15105900	-6.76888900	-2.57386800
C	-0.15809200	-7.10811400	-2.30116500
C	-2.91817300	-10.00466600	3.10200400
C	-10.38998200	-3.16724200	-2.66362300
C	-4.52344800	-8.33474800	4.96467500
C	-9.15716200	-5.20976100	-4.43819500
C	-10.27965700	-4.28770000	-4.94463300
C	-11.19556100	-3.82004800	-3.80030000
C	-3.35007400	-10.54229300	4.47692100
C	-3.54752300	-9.40148100	5.48986200
C	2.84603600	-5.26238900	-3.53432200
C	3.52206600	-4.48538700	2.43898300
C	2.94483200	-4.43123000	3.72751100
C	1.61761200	-4.77789700	3.92251600
N	0.83316500	-5.16252800	2.87915200
C	1.35711800	-5.22581900	1.62117100
C	2.68046200	-4.90806400	1.38078600
C	7.81140900	-1.27388700	-2.60869000
C	7.02421800	-0.21088400	-3.07104400
C	7.06412800	1.05884700	-2.44156600
C	7.91296500	1.22580800	-1.33009600
C	8.68915700	0.16224500	-0.83983200
C	8.65885300	-1.07329100	-1.49958000
C	7.66747200	-2.62153200	-3.19975000
N	8.38848600	-3.59831800	-2.79728000
C	9.42630400	0.29035700	0.43267400
N	9.09656800	1.16756500	1.30871900
C	9.78699000	1.09358800	2.59375300
C	8.12916700	-4.92879700	-3.32308000
C	6.77155000	-2.55828700	2.65105900
C	5.46517800	-2.99214300	2.90506800

C	4.91853700	-4.08818800	2.19936700
C	5.69870600	-4.74589500	1.23208800
C	7.02408700	-4.33687700	0.98871700
C	7.55401400	-3.25873900	1.71184900
C	7.27238100	-1.31630100	3.27055800
N	8.46662400	-0.90106400	3.08018600
C	7.83848200	-4.93109600	-0.09031600
N	7.32048200	-5.54586100	-1.09053000
C	8.24729700	-5.94459900	-2.14947800
C	8.84295800	0.39819100	3.61667500
C	2.15902000	-3.44995800	-1.85406800
C	2.49753900	-2.46153600	-0.92547800
C	3.84112700	-2.21746900	-0.57470200
C	4.83212300	-3.02059100	-1.17703000
C	4.49625000	-4.03097700	-2.07993200
C	3.15562500	-4.24415400	-2.44634400
C	4.19129700	-1.15318700	0.39659400
C	5.39498400	-0.43267600	0.27929900
C	5.70199700	0.61443400	1.15438600
C	4.80320800	0.96897700	2.17270200
C	3.62227700	0.22132600	2.33636100
C	3.32680500	-0.82991500	1.46648900
C	5.09227900	2.14853700	3.07223500
C	6.15009000	2.14347000	-2.84060900
C	5.10511500	1.94965000	-3.77726600
C	4.17024500	2.93869800	-4.02037500
N	4.22683100	4.13425700	-3.37024200
C	5.25881400	4.39020900	-2.51746700
C	6.22274800	3.43736300	-2.25928100
C	10.19670000	2.48297300	3.10291300
C	7.95936000	-7.36657800	-2.65216200
C	9.56592400	0.25953400	4.97349200
C	9.14214900	-5.29649500	-4.42909700
C	8.90439600	-6.73263500	-4.92719300
C	8.93948700	-7.75068800	-3.77427300
C	10.90218400	2.36860000	4.46512500
C	10.02567600	1.63269500	5.49314900
C	3.08977600	5.08406500	-3.45657600
C	2.19922300	5.28337100	2.42032300
C	2.42614800	4.74604300	3.70752000
C	3.37010100	3.75058600	3.90215800
N	4.09268500	3.25650100	2.86000400
C	3.90659100	3.75828600	1.60514900
C	2.98842800	4.76305300	1.36569800

C	-2.79321300	7.35941800	-2.61884100
C	-3.34069000	6.15398200	-3.07815200
C	-4.46742000	5.57184900	-2.44396200
C	-5.02251200	6.23419400	-1.33183800
C	-4.46622900	7.42763000	-0.84181900
C	-3.37423000	8.00135500	-1.50575800
C	-1.55347700	7.89512300	-3.22145000
N	-1.04881100	8.99874300	-2.81728700
C	-4.92724400	8.00492400	0.43610900
N	-5.52868300	7.28592500	1.31189600
C	-5.78167700	7.91406900	2.60576700
C	0.22517600	9.43629400	-3.36547700
C	-1.09583600	7.12954100	2.61578700
C	-0.06294400	6.22138000	2.87678500
C	1.16203400	6.29849900	2.17612100
C	1.33996500	7.30003300	1.20466300
C	0.31887900	8.23648200	0.95333500
C	-0.88188900	8.15449000	1.67376900
C	-2.42045300	6.93758000	3.23635500
N	-3.38225400	7.75866900	3.04626500
C	0.41627700	9.22941500	-0.13556900
N	1.19857900	9.08099000	-1.14181400
C	1.06060200	10.06727000	-2.21319100
C	-4.68749200	7.43332900	3.60226300
C	1.89420300	3.58099300	-1.75074400
C	0.87036600	3.37571700	-0.82010400
C	-0.03967100	4.40296800	-0.49771700
C	0.13708100	5.65551100	-1.12200200
C	1.17801200	5.87531700	-2.02466000
C	2.05628000	4.83265900	-2.36824900
C	-1.14616700	4.17280200	0.46245500
C	-2.37984800	4.83362600	0.31024600
C	-3.44901200	4.58370600	1.17604100
C	-3.30910900	3.65574000	2.22002300
C	-2.06500200	3.02806900	2.41730600
C	-0.99812500	3.29054100	1.55579100
C	-4.48517200	3.32013900	3.10758400
C	-4.97065300	4.24350600	-2.83664300
C	-4.30028500	3.42502500	-3.77886500
C	-4.70576100	2.12387700	-4.01201200
N	-5.76770300	1.58883900	-3.34762500
C	-6.48804600	2.36587100	-2.49034600
C	-6.12767700	3.67414500	-2.24092900
C	-7.17947100	7.57380700	3.14226400

C	2.42616600	10.52817100	-2.74260100
C	-4.89957500	8.11607200	4.97064200
C	0.01660400	10.48061900	-4.48419600
C	1.36923400	10.99171100	-5.00950600
C	2.24744400	11.55178500	-3.87724500
C	-7.40313500	8.22990000	4.51536800
C	-6.30786400	7.82542000	5.51693700
H	-7.11984200	-0.02147200	-3.34365600
H	-5.73028300	-0.20874300	-4.42116900
H	-5.40716100	-0.95366900	4.61823200
H	-4.72342500	1.41918700	4.92208000
H	-5.18921200	2.22930400	0.85616500
H	-5.91804600	-0.11687200	0.38604300
H	-3.54621300	-5.28288700	-3.94165600
H	-2.12832500	-7.96457100	-0.80642100
H	-6.29873000	-6.98364700	-1.12566300
H	-5.82351500	-4.52437100	-3.95947100
H	-5.51321600	-8.56433400	0.62576000
H	-4.93453300	-9.44082300	2.52337300
H	-7.90225300	-3.58885700	-3.73946300
H	-4.57421900	-2.93244500	3.60658600
H	-7.57873200	-1.76062400	0.69282000
H	-6.83239900	-5.93150800	1.51490200
H	-3.92157900	-5.22238500	3.83490700
H	-8.62018400	-5.32297900	-0.02148200
H	-9.78017500	-5.06931700	-1.81024700
H	-3.05537500	-7.32991300	3.72037400
H	-3.73183000	0.85154900	-1.96899500
H	-2.50531900	-0.51885400	-0.35907700
H	-5.41253900	-3.69004500	-0.83007300
H	-6.68928600	-2.30353100	-2.37182500
H	-3.57438200	-4.93367100	-0.48881600
H	-2.26957800	-6.34772900	1.06652200
H	-1.06946200	-2.81084300	3.25001900
H	-2.47361900	-1.41831800	1.76769900
H	-0.73433000	-6.58312500	2.88139800
H	-0.81918100	-5.35460200	4.16200700
H	-1.62967500	-4.82702700	-4.37171900
H	0.74724400	-4.33205300	-4.79284500
H	1.99902300	-7.28089800	-2.11820000
H	-0.32516200	-7.94716000	-1.62717700
H	-2.82845200	-10.82235700	2.36796100
H	-1.92101200	-9.53084700	3.16955800
H	-11.04722200	-2.88424300	-1.82492300

H	-9.91042000	-2.23542800	-3.01748600
H	-4.60582800	-7.49133900	5.67099900
H	-5.53851600	-8.75572000	4.85275100
H	-9.57373900	-6.15359100	-4.04376500
H	-8.46944100	-5.48139300	-5.25677100
H	-10.86612800	-4.81238000	-5.71701500
H	-9.83162200	-3.40633100	-5.44070200
H	-11.75505700	-4.68596600	-3.40004200
H	-11.95028700	-3.11153000	-4.17960200
H	-4.29567900	-11.10541000	4.36830100
H	-2.60210400	-11.26103100	4.85014000
H	-2.57043900	-8.92899600	5.70460900
H	-3.91666100	-9.79824200	6.45005300
H	3.52002100	-6.12953800	-3.47926200
H	2.96938700	-4.82197300	-4.53555300
H	3.53930600	-4.15131500	4.59863100
H	1.14910500	-4.76850800	4.90681600
H	0.66200700	-5.51412100	0.83113800
H	3.05194600	-4.94919000	0.35536900
H	6.37602200	-0.39165400	-3.93190500
H	7.96677800	2.16357100	-0.77426300
H	9.24900900	-1.92107700	-1.13882700
H	6.86696800	-2.74218600	-3.95940500
H	10.22377000	-0.45530100	0.61385100
H	10.69047200	0.45110200	2.51748000
H	7.10488700	-5.00264100	-3.75345600
H	4.84405000	-2.42845800	3.60737500
H	5.30501600	-5.58901600	0.65921200
H	8.57425500	-2.90837500	1.52730000
H	6.52882000	-0.73781400	3.85938300
H	8.92970300	-4.75315100	-0.01638500
H	9.29911800	-5.88831300	-1.79432700
H	7.94751500	1.04682100	3.75884900
H	1.10472000	-3.58100800	-2.11339700
H	1.70159500	-1.84638900	-0.49857500
H	5.88204900	-2.89228600	-0.90987500
H	5.29468400	-4.67393800	-2.46000900
H	6.09528900	-0.68023300	-0.51901000
H	6.66502500	1.13146900	1.07007900
H	2.93639400	0.45600400	3.15568300
H	2.43347900	-1.43620700	1.64006700
H	6.09248000	2.56642000	2.88374700
H	5.03535400	1.88094700	4.13632300
H	4.98386700	1.01208000	-4.31799800

H	3.33824700	2.80205800	-4.71178600
H	5.27371700	5.38069100	-2.06222900
H	7.03999100	3.72103900	-1.59740800
H	10.85175600	2.96409200	2.35792700
H	9.29494300	3.11769500	3.18701000
H	8.02928500	-8.07135700	-1.80724100
H	6.91688700	-7.41450200	-3.01830600
H	8.88407800	-0.22725900	5.69121900
H	10.42875600	-0.41806200	4.84492700
H	10.16405500	-5.18683200	-4.02462800
H	9.04550400	-4.57101400	-5.25439100
H	9.65999100	-6.98746900	-5.68874600
H	7.92279100	-6.78523000	-5.43439200
H	9.96406700	-7.80313100	-3.36128000
H	8.70368900	-8.76063400	-4.14832600
H	11.85589100	1.82337700	4.33752600
H	11.16611000	3.37274100	4.83579300
H	9.13866600	2.25079900	5.72733400
H	10.57162300	1.50344200	6.44232200
H	3.49498800	6.10494500	-3.39832800
H	2.64540700	4.97188200	-4.45730200
H	1.88761400	5.12430800	4.57783300
H	3.58109200	3.32868900	4.88502300
H	4.50218100	3.29649200	0.81597500
H	2.84985200	5.11386000	0.34168900
H	-2.87117800	5.67461000	-3.94058400
H	-5.86831800	5.82797500	-0.77447200
H	-2.91818300	8.92884200	-1.14657800
H	-1.06937500	7.26250200	-3.99481900
H	-4.65865400	9.06211200	0.62219400
H	-5.67500400	9.01785700	2.53467400
H	0.79837800	8.58254100	-3.79270100
H	-0.24035700	5.40343900	3.58112000
H	2.26750400	7.38125300	0.63275700
H	-1.69810300	8.85803600	1.48264200
H	-2.54269100	6.00685300	3.83018200
H	-0.28725800	10.08260500	-0.06512000
H	0.48672800	10.95230600	-1.86238100
H	-4.80071800	6.33247100	3.73622400
H	2.55643800	2.74390900	-1.98926200
H	0.76100100	2.38686100	-0.36766800
H	-0.51671000	6.49182500	-0.86970900
H	1.32030600	6.88471400	-2.41947500
H	-2.51385800	5.54160200	-0.50818700

H	-4.38168700	5.14935200	1.06716200
H	-1.92685500	2.34124500	3.25777400
H	-0.02350000	2.83633000	1.75335800
H	-5.34731000	3.97182700	2.90166200
H	-4.23999300	3.41371100	4.17446100
H	-3.43062600	3.77748700	-4.33176500
H	-4.18524000	1.46260900	-4.70543700
H	-7.35255900	1.89359400	-2.02287400
H	-6.76768500	4.24877600	-1.57265800
H	-7.93871900	7.90768200	2.41591500
H	-7.27855000	6.47483000	3.21928700
H	3.01163300	10.95623100	-1.91225700
H	2.98798000	9.64683000	-3.10419900
H	-4.12268200	7.76129400	5.66897100
H	-4.74488900	9.20301200	4.84916300
H	-0.58723400	11.31382800	-4.08283100
H	-0.57348600	10.01966000	-5.29421400
H	1.19711300	11.76176500	-5.77971700
H	1.90198500	10.16325500	-5.51288500
H	1.78230500	12.46912900	-3.47080000
H	3.23293600	11.85163200	-4.27016600
H	-7.40648800	9.32960500	4.39828500
H	-8.39781800	7.95624100	4.90399400
H	-6.39760000	6.74586800	5.74140900
H	-6.44775400	8.35296700	6.47494700

***P*-*A*-*R*-2**$^{6+}$ (ΔG = 17.6 kcal/mol)
Total SCF energy (BP86-D3/6-311G(d)/PCM(water)): -7021.246530 a.u.
Thermal correction to Gibbs free energy at 298.15 K: 2.475032 a.u.
Gibbs free energy at 298.15 K (BP86-D3/6-311G(d)/PCM(water)): -7018.771498 a.u.

C	0.79647500	-1.89791900	3.60058500
C	4.83995600	-1.76886900	2.02845000
C	4.48798800	-1.19843700	3.28038900
C	3.18993200	-1.23895700	3.74997600
N	2.19038500	-1.81492000	3.02057700
C	2.48201500	-2.35523400	1.80463500
C	3.76523800	-2.33647100	1.29900800
C	7.42757700	2.95794500	-2.68445600
C	6.71872200	2.33187100	-3.73969900
C	5.68525500	2.98435100	-4.38469800
N	5.31504400	4.24347100	-4.01904900
C	6.00172500	4.89822000	-3.03970600

C	7.05257000	4.29215500	-2.37994900
C	4.06061600	4.83854300	-4.55370900
C	-0.48653000	-5.53211700	-2.55151400
C	-0.87673400	-4.50342000	-3.43565300
C	0.08194700	-3.72032500	-4.05730500
N	1.41059500	-3.93557600	-3.84108500
C	1.81915300	-4.99178900	-3.08175100
C	0.90037600	-5.80378200	-2.45009100
C	3.01527000	2.23226000	1.87667400
C	2.89813300	1.12151100	1.04458600
C	3.69160800	0.98886000	-0.11720200
C	4.66435400	1.97867500	-0.36118800
C	4.77488700	3.10128800	0.46696400
C	3.93307300	3.25593600	1.57856400
C	3.93919700	4.52190100	2.41199400
C	3.41934000	-0.09010300	-1.09720400
C	2.10233100	-0.57763700	-1.25387400
C	1.78945800	-1.51949700	-2.23257400
C	2.78181900	-2.00824600	-3.10127300
C	4.10533900	-1.57024700	-2.92115600
C	4.41892400	-0.62240700	-1.93810500
C	2.42375400	-2.92708400	-4.26102000
C	-0.52181600	-0.92890400	1.67492300
C	-1.32034900	-1.08751100	0.54248100
C	-1.85962300	-2.35286500	0.20305500
C	-1.65491500	-3.41543900	1.10678100
C	-0.85336600	-3.25733400	2.24110800
C	-0.23768600	-2.02431300	2.51290600
C	-2.63504900	-2.53448600	-1.04828700
C	-2.44250500	-1.65866100	-2.14320100
C	-3.26872600	-1.71519400	-3.26420000
C	-4.30864500	-2.66296200	-3.33981000
C	-4.42653800	-3.62100300	-2.31966000
C	-3.60025900	-3.55340100	-1.18947100
C	-0.03957600	6.10658000	1.97700900
C	0.47318600	5.75186100	3.24764500
C	1.77369300	5.29531000	3.38790200
N	2.59869400	5.18466700	2.31003900
C	2.16546600	5.59108800	1.08292800
C	0.88445800	6.06234200	0.90352000
C	-6.98847700	0.98279800	-2.52762300
C	-6.21926700	1.08844800	-3.71107600
C	-5.82569500	-0.04551400	-4.40006500
N	-6.18690700	-1.28753400	-3.97087600

C	-7.05933400	-1.41236600	-2.93168500
C	-7.48262400	-0.31071400	-2.21537200
C	-5.38991300	-2.49332500	-4.39352000
C	-3.80693300	6.32652100	2.43372000
C	-2.44779700	6.06261300	2.68431100
C	-1.45749600	6.43534300	1.75054200
C	-1.86639200	7.04272000	0.54130100
C	-3.21833500	7.27240300	0.25916000
C	-4.18154300	6.94646600	1.23182700
C	-4.87245600	5.91745500	3.37517100
C	-3.62139500	7.71809200	-1.09075600
C	-7.22033600	4.53275000	-1.18038000
C	-7.11983600	3.45651200	-2.08449600
C	-7.18329300	2.12737400	-1.62265000
C	-7.31692700	1.88707400	-0.23135000
C	-7.34725700	2.94801500	0.68204800
C	-7.33381400	4.27003100	0.19155600
C	-7.16831700	5.93789200	-1.63430100
C	-7.22618900	2.68966900	2.13254800
C	-1.46549200	2.99722900	0.99953100
C	-0.37030100	3.20990700	0.16624600
C	-0.51815200	3.90015200	-1.06032900
C	-1.80034100	4.36066900	-1.41722600
C	-2.90299700	4.13480300	-0.58554200
C	-2.74291500	3.46052900	0.63210400
C	-3.92236900	3.19475800	1.52583800
C	0.65625000	4.15143200	-1.92682000
C	1.70147400	3.21097100	-2.00963700
C	2.80123800	3.43217900	-2.83678800
C	2.90390900	4.61104000	-3.59422500
C	1.88191300	5.56945800	-3.49829000
C	0.77160200	5.33765100	-2.68188000
C	-4.81664100	-1.00762000	1.60333900
C	-4.57010200	-0.29192800	2.79625200
C	-4.27989600	1.06242900	2.75398500
N	-4.21668000	1.72609200	1.56808100
C	-4.41638100	1.06124800	0.39711400
C	-4.70759900	-0.28759500	0.38839100
N	-4.77920400	4.88524700	4.13202200
C	-5.95902500	4.58458500	4.94809800
N	-7.00159200	3.65554800	2.94144700
C	-6.72319200	3.37496400	4.34420900
C	-5.57952300	4.29691500	6.40869500
C	-8.01277000	3.12367000	5.15858900

C	-6.84234000	4.01384100	7.24154300
C	-7.67662000	2.87046500	6.63853000
N	-6.70964400	6.26468300	-2.78425600
C	-6.63801300	7.69634400	-3.06447500
N	-4.85159000	7.87586400	-1.40108100
C	-5.18947300	8.18964200	-2.78767900
C	-7.03828900	7.99798700	-4.51672600
C	-5.10257700	9.71000900	-3.05209200
C	-6.93591600	9.50481900	-4.80551400
C	-5.52868200	10.04128000	-4.49239900
C	-5.07963900	-4.68321600	2.51944400
C	-4.68810500	-3.33020200	2.55118300
C	-5.20051900	-2.42489500	1.60506400
C	-6.09445300	-2.88930100	0.61640600
C	-6.47998400	-4.23281900	0.56371300
C	-5.97632500	-5.12203100	1.53163900
C	-4.54868200	-5.67451800	3.47360200
C	-7.24536000	-4.70710100	-0.59966500
C	-3.63933500	-7.15827900	-1.16994300
C	-2.75520500	-6.55016700	-2.08364800
C	-1.44650400	-6.22724000	-1.68245000
C	-1.04482300	-6.48322400	-0.35392900
C	-1.92770200	-7.04233400	0.57707700
C	-3.21887000	-7.40506800	0.14888000
C	-5.02344200	-7.51816100	-1.53402800
C	-1.52630400	-7.07466600	1.99574100
N	-3.53462200	-5.45900700	4.22794600
C	-3.09680600	-6.61225100	5.01572700
N	-2.27616600	-7.53318000	2.92275300
C	-1.87753300	-7.26740900	4.29679300
C	-2.71415700	-6.23274600	6.45247100
C	-1.46708900	-8.54728800	5.04925600
C	-2.26186300	-7.48323900	7.22836000
C	-1.11386800	-8.21573800	6.51076800
N	-5.66032500	-7.00277600	-2.52165900
C	-7.05453400	-7.43597700	-2.65022600
N	-7.52510700	-5.93425900	-0.80780800
C	-7.99044500	-6.30102100	-2.13521700
C	-7.42622700	-7.80166000	-4.09390000
C	-9.44813000	-6.79713400	-2.14588500
C	-8.89231700	-8.26850300	-4.15767300
C	-9.85365600	-7.21948300	-3.57024900
C	10.14891800	0.48043200	-1.79094500
C	9.16819200	1.17924900	-2.51653700

C	8.47187300	2.25242700	-1.93207500
C	8.75586900	2.60801300	-0.58971000
C	9.73359800	1.92416300	0.14235000
C	10.44059200	0.87172800	-0.47702600
C	10.75850400	-0.74431700	-2.34539600
C	9.92572900	2.21430100	1.57835100
C	8.62856300	-1.53923300	1.93323600
C	7.29554200	-1.39965600	2.35706100
C	6.22545600	-1.81138100	1.53659800
C	6.52409600	-2.31879900	0.24461000
C	7.84597400	-2.47389800	-0.18597700
C	8.89556400	-2.10263700	0.67841700
C	9.74979000	-1.02589300	2.74407800
C	8.14529800	-2.95299400	-1.55166200
N	10.31290900	-1.28181800	-3.41968900
C	10.76392600	-2.63376100	-3.71370200
N	9.32058600	-3.32597600	-1.88684200
C	9.60059800	-3.59288500	-3.28738000
C	11.08997200	-2.83768200	-5.19969600
C	10.02634200	-5.05424700	-3.52003700
C	11.48506600	-4.30047000	-5.46871800
C	10.39394500	-5.27831600	-4.99766100
N	9.56512200	-0.26299100	3.75648800
C	10.75556800	0.37046000	4.31100400
N	10.85943200	1.66213800	2.25590600
C	10.84257100	1.81234800	3.70164000
C	10.71240100	0.45112700	5.84297100
C	12.11362700	2.51167500	4.21850600
C	11.95660600	1.17807100	6.38233300
C	12.11039000	2.57520000	5.75586900
H	0.65786700	-0.98815600	4.20037200
H	0.78179000	-2.75898800	4.28402100
H	5.23112900	-0.72299400	3.91942400
H	2.90686400	-0.82890300	4.72004200
H	1.65042700	-2.79901300	1.25978200
H	3.91625500	-2.79670400	0.32369000
H	6.95428200	1.31447200	-4.05350000
H	5.10835000	2.52525100	-5.18755800
H	5.67566200	5.91619200	-2.82454800
H	7.60519200	4.87789400	-1.64426000
H	3.86847400	4.37659300	-5.53388500
H	4.23992300	5.90837600	-4.73039100
H	-1.92997500	-4.26347600	-3.59095500
H	-0.17198600	-2.88166100	-4.70532200

H	2.89594600	-5.13721200	-2.99764100
H	1.26538200	-6.65218500	-1.86927900
H	2.37742200	2.31532300	2.76189800
H	2.17852800	0.33909000	1.29658200
H	5.30633400	1.90987000	-1.24331800
H	5.50780000	3.87692300	0.22369400
H	4.69696900	5.24069000	2.07194800
H	4.12501000	4.32097600	3.47590400
H	1.29417300	-0.19077000	-0.62665700
H	0.75071400	-1.85302400	-2.32608800
H	4.90092300	-1.96015200	-3.56299400
H	5.46101000	-0.31486200	-1.80890100
H	3.30109600	-3.46468600	-4.64631900
H	1.99917600	-2.35878400	-5.10194800
H	-0.10832500	0.05861600	1.90794100
H	-1.54215700	-0.20868200	-0.07001600
H	-2.09586100	-4.39424100	0.91337200
H	-0.70211100	-4.10937500	2.91071900
H	-1.64211300	-0.91501200	-2.11764200
H	-3.11264700	-1.00296300	-4.07933700
H	-5.16073600	-4.43189000	-2.39460000
H	-3.75857400	-4.28260100	-0.39188500
H	-0.13451900	5.82716200	4.14973200
H	2.19006700	5.00304300	4.35220700
H	2.86400300	5.47026400	0.25476200
H	0.60020300	6.33863900	-0.10954200
H	-5.86134900	2.05375500	-4.07100600
H	-5.17898800	-0.00209300	-5.27655200
H	-7.36609200	-2.42992700	-2.68578900
H	-8.19983500	-0.46158600	-1.40699000
H	-4.99860500	-2.29658200	-5.40028600
H	-6.06935400	-3.35430000	-4.44942800
H	-2.19648000	5.54225000	3.61184300
H	-1.12861300	7.34890900	-0.20481100
H	-5.23364100	7.14711000	1.00651300
H	-5.78808700	6.53950600	3.35055800
H	-2.79879600	7.83367300	-1.82918900
H	-7.02998000	3.69636900	-3.14751600
H	-7.33005700	0.86402300	0.15467300
H	-7.38005900	5.08964700	0.91510300
H	-7.52399000	6.68856100	-0.90138200
H	-7.24484300	1.62525200	2.45136900
H	-1.33178300	2.49017900	1.96014800
H	0.62460400	2.88538900	0.48767000

H	-1.95003700	4.86998100	-2.37262800
H	-3.88994400	4.50569700	-0.88211500
H	-4.83009300	3.70113600	1.16740100
H	-3.77389500	3.52357100	2.56633700
H	1.63969200	2.27112900	-1.45601300
H	3.57070400	2.65706300	-2.89286900
H	1.94404200	6.50105500	-4.06786100
H	-0.00878700	6.10212700	-2.62664700
H	-4.64686700	-0.78010200	3.76888600
H	-4.10519900	1.66346000	3.64716200
H	-4.31904100	1.65713800	-0.51040800
H	-4.83101300	-0.78561000	-0.57494700
H	-6.66890400	5.43924800	4.92288400
H	-6.08403600	2.46893500	4.43740600
H	-4.89015600	3.43289300	6.44149300
H	-5.02462500	5.15758700	6.81643100
H	-8.67440800	4.00153900	5.05189100
H	-8.55122500	2.26469800	4.72351000
H	-6.55893800	3.77252800	8.27902200
H	-7.45800900	4.93086500	7.29263500
H	-8.61047500	2.73418800	7.20827100
H	-7.11709300	1.92061800	6.72760500
H	-7.29685000	8.26670400	-2.37379100
H	-4.50383600	7.66619400	-3.48644400
H	-6.37763300	7.42626800	-5.19377500
H	-8.06341000	7.63157900	-4.69220700
H	-5.74851700	10.23375300	-2.32461000
H	-4.06921300	10.04624900	-2.86015800
H	-7.19534300	9.70308700	-5.85865600
H	-7.68077800	10.04783300	-4.19399500
H	-5.48825100	11.13258000	-4.64598900
H	-4.80125100	9.59831700	-5.19812300
H	-3.95754100	-3.02231900	3.30426700
H	-6.50027200	-2.19201900	-0.12286200
H	-6.27865700	-6.17243000	1.47886600
H	-5.07516500	-6.64943700	3.46995000
H	-7.46758800	-3.92882700	-1.36492200
H	-3.09913300	-6.37012000	-3.10556400
H	-0.05073500	-6.17593000	-0.01540100
H	-3.90094500	-7.85233600	0.87856500
H	-5.50334900	-8.25409900	-0.85905100
H	-0.55422400	-6.58281600	2.22022300
H	-3.89240300	-7.38776700	5.04492800
H	-1.03034600	-6.54523100	4.33501500

H	-1.90800700	-5.47724400	6.42636600
H	-3.57885500	-5.75629500	6.94357700
H	-2.29784700	-9.27344800	5.00086600
H	-0.60869100	-9.00746800	4.53170400
H	-1.95482900	-7.19947900	8.24855100
H	-3.12091800	-8.17050600	7.34247800
H	-0.85681600	-9.14389200	7.04759200
H	-0.20600500	-7.58415000	6.53091600
H	-7.24640700	-8.31384900	-1.99660100
H	-7.90274900	-5.43857800	-2.83865700
H	-7.26680800	-6.92384700	-4.74640000
H	-6.74602800	-8.59123600	-4.45379700
H	-9.54259400	-7.64344500	-1.44280000
H	-10.10507500	-5.99484200	-1.77056800
H	-9.16686900	-8.49464500	-5.20109300
H	-8.99573700	-9.21606200	-3.59700700
H	-10.88482800	-7.60922900	-3.55881100
H	-9.86732700	-6.32774100	-4.22424300
H	8.98560300	0.85735700	-3.54507800
H	8.18262800	3.39537900	-0.09054500
H	11.19204800	0.34007000	0.11481600
H	11.54502000	-1.21888800	-1.72452200
H	9.16939300	2.87886800	2.04602500
H	7.14457100	-0.96433800	3.34752300
H	5.72574000	-2.61088800	-0.44196700
H	9.92360100	-2.23840700	0.32974300
H	10.76251200	-1.27878700	2.37060500
H	7.30390200	-2.91505400	-2.27908600
H	11.64637200	-2.91097100	-3.09610200
H	8.72360100	-3.36091600	-3.93174100
H	10.20768600	-2.55463600	-5.80203700
H	11.90385000	-2.15087400	-5.48669900
H	10.88467300	-5.28475600	-2.86407400
H	9.20226000	-5.72058800	-3.21389200
H	11.68729100	-4.44182100	-6.54348100
H	12.43129900	-4.52618100	-4.94197300
H	10.72358900	-6.32062900	-5.14292800
H	9.49012700	-5.14779400	-5.62211200
H	11.67901100	-0.16035800	3.99139500
H	9.94771300	2.37664200	4.04502000
H	9.79076000	0.97957400	6.14696100
H	10.64335700	-0.56981800	6.25479700
H	12.99791500	1.95987100	3.85239500
H	12.16602400	3.52377900	3.78285700

H	11.89872700	1.25491500	7.48092500
H	12.85748400	0.57569000	6.15974200
H	13.03857100	3.05502300	6.10890100
H	11.27802800	3.22145100	6.09255700

***P*-*Ā*-*R*-2**$^{6+}$ (ΔG = 25.6 kcal/mol)
Total SCF energy (BP86-D3/6-311G(d)/PCM(water)): -7021.236596 a.u.
Thermal correction to Gibbs free energy at 298.15 K: 2.477859 a.u.
Gibbs free energy at 298.15 K (BP86-D3/6-311G(d)/PCM(water)): -7018.758737 a.u.

C	-0.53327600	-5.73915600	-3.93627500
C	-4.46630400	-4.71541900	-2.52635800
C	-3.86590900	-4.04263600	-3.61785800
C	-2.63389600	-4.43490100	-4.10652100
N	-1.94651200	-5.46406200	-3.53810500
C	-2.50573700	-6.16149300	-2.50945300
C	-3.74865800	-5.82595200	-2.01218300
C	-5.40669600	2.61138100	2.24921300
C	-4.14287500	2.25545600	2.77633700
C	-3.11800600	3.17922300	2.83038400
N	-3.29959100	4.45591000	2.38882200
C	-4.50673900	4.84031400	1.89099500
C	-5.56257200	3.94803700	1.81258100
C	-2.13617500	5.38391200	2.38332600
C	0.43951800	-5.98218500	2.27579700
C	0.11441700	-4.71418000	2.81227900
C	-1.19892500	-4.29068800	2.87080200
N	-2.21251800	-5.08296000	2.41966200
C	-1.94051200	-6.31880900	1.91756700
C	-0.63963000	-6.78622200	1.83919500
C	-7.80243000	-2.95814600	-2.09449100
C	-6.60928600	-3.41588800	-2.66287300
C	-5.74109100	-4.27394100	-1.94315600
C	-6.09214300	-4.65068300	-0.63274600
C	-7.28784500	-4.19978100	-0.04772700
C	-8.14073900	-3.37282700	-0.79004200
C	-8.64663000	-1.96927000	-2.80038300
N	-9.68434900	-1.49813200	-2.21705400
C	-7.61425300	-4.42408000	1.37469700
N	-6.72762100	-4.78902500	2.22737100
C	-7.02405900	-4.75046100	3.66056500
C	-10.41178800	-0.36011300	-2.77946500
C	-7.59812500	-0.40775200	2.92913700

C	-6.62868500	0.58959000	3.09413500
C	-6.49045800	1.62309100	2.13412500
C	-7.34605500	1.65509000	1.01857600
C	-8.33270700	0.66555800	0.85073700
C	-8.45486700	-0.34812800	1.81039600
C	-7.64567200	-1.58654400	3.82973100
N	-8.27339700	-2.62784700	3.43129500
C	-9.14909200	0.56843000	-0.37765300
N	-8.84232100	1.20930600	-1.44473400
C	-9.54049200	0.93575800	-2.70123600
C	-8.25177100	-3.89988300	4.14276100
C	-3.33173000	1.59921900	-2.71962700
C	-3.20805400	0.47060800	-1.91080500
C	-4.34750000	-0.16577500	-1.37249800
C	-5.61263000	0.38292000	-1.66274000
C	-5.73563700	1.53279900	-2.45130800
C	-4.59728100	2.14487200	-2.99960700
C	-4.73401500	3.34532600	-3.91929200
C	-4.18843400	-1.33028500	-0.46941400
C	-3.16584000	-2.28007000	-0.66366600
C	-2.96153400	-3.31592700	0.25221800
C	-3.76196800	-3.41764200	1.40147400
C	-4.79105900	-2.48095800	1.59708400
C	-5.01354000	-1.46834900	0.66541300
C	-3.59775400	-4.53840100	2.40886000
C	6.47436100	-5.27745500	-2.10768700
C	6.28276000	-4.01379600	-2.67593700
C	6.59359700	-2.83463300	-1.95386100
C	7.09200300	-2.95451500	-0.64254400
C	7.29315200	-4.21694100	-0.05869600
C	6.99936300	-5.36693100	-0.80224600
C	6.03302100	-6.50042100	-2.81390700
N	6.12266300	-7.63337500	-2.22452500
C	7.64823000	-4.38928600	1.36398800
N	7.52618800	-3.43733400	2.21546300
C	7.64236200	-3.71088600	3.64866300
C	5.48965300	-8.82619600	-2.78792600
C	4.15605100	-6.36340600	2.92979200
C	2.80827800	-6.02481700	3.10476700
C	1.83786100	-6.42230100	2.15134100
C	2.23169200	-7.17587600	1.03116400
C	3.58114200	-7.53364600	0.85313400
C	4.52605500	-7.13329900	1.80735400
C	5.20533900	-5.81603200	3.82550300

N	6.42103200	-5.85130600	3.42802000
C	4.06650400	-8.18908600	-0.37995900
N	3.35387700	-8.23810700	-1.44455700
C	3.93284300	-8.70287900	-2.70552000
C	7.51507600	-5.19740400	4.13553800
C	0.29384400	-3.65112200	-2.74907500
C	1.21115600	-2.98460400	-1.93815200
C	2.31875600	-3.66248100	-1.38424700
C	2.46117400	-5.03661800	-1.66147300
C	1.52464500	-5.71316700	-2.45139900
C	0.43904600	-5.02448300	-3.01529200
C	3.25197000	-2.94771000	-0.48155900
C	3.57776600	-1.59127500	-0.68028500
C	4.37788500	-0.90162800	0.23467500
C	4.86134800	-1.54608500	1.38471300
C	4.55075300	-2.90158100	1.58493800
C	3.77562200	-3.59460900	0.65671200
C	-1.86851200	6.23417800	-2.52372700
C	-1.59281100	5.37705300	-3.61641200
C	-2.55290600	4.50830600	-4.10048500
N	-3.78562400	4.43035000	-3.52641100
C	-4.10281000	5.26339200	-2.49556800
C	-3.18666500	6.17021100	-2.00296100
C	4.98510000	3.37084500	2.26750500
C	4.05251300	2.45333100	2.80573000
C	4.34036500	1.10381500	2.85539900
N	5.53340800	0.62434800	2.40216200
C	6.46514700	1.47955400	1.89829700
C	6.21822900	2.83970300	1.82150000
C	5.75658000	-0.84710300	2.38766800
C	1.32945700	8.23354200	-2.09640700
C	0.33134500	7.43607400	-2.66592200
C	-0.84420000	7.11365500	-1.94315100
C	-0.98882300	7.60107100	-0.63034600
C	0.00354000	8.40595100	-0.04537900
C	1.14483500	8.72979500	-0.78969600
C	2.60841100	8.46862900	-2.80234700
N	3.53945300	9.12035300	-2.21314900
C	-0.02329800	8.79560500	1.37829800
N	-0.78441200	8.20951100	2.22870300
C	-0.60594300	8.44273100	3.66240700
C	4.88855300	9.18157000	-2.77540600
C	3.44442100	6.77062600	2.93634700
C	3.83257000	5.43573100	3.10615200

C	4.66280700	4.80117600	2.14867200
C	5.11201500	5.52447500	1.02958000
C	4.74131900	6.87126200	0.85766400
C	3.92127500	7.48230900	1.81575300
C	2.44286100	7.39694000	3.83518300
N	1.85871300	8.46495800	3.44113300
C	5.06123000	7.62537600	-0.37293600
N	5.46616900	7.03928500	-1.43887300
C	5.57509100	7.77883700	-2.69699500
C	0.74323600	9.07863300	4.15119700
C	3.04399300	2.07738800	-2.74632700
C	2.00760200	2.53299200	-1.93295600
C	2.03442300	3.83186200	-1.38069500
C	3.14735700	4.64915100	-1.66154200
C	4.20322100	4.18137500	-2.45235700
C	4.15593200	2.89582500	-3.01469000
C	5.26372100	2.41477300	-3.93452500
C	0.94683100	4.27596300	-0.47722200
C	-0.38864500	3.87438600	-0.67908300
C	-1.39007200	4.22001700	0.23215500
C	-1.07898300	4.96088600	1.38371600
C	0.24852500	5.37315400	1.58822500
C	1.23989000	5.05295300	0.66213000
C	6.34158700	-1.50777700	-2.53402100
C	5.46769900	-1.32062900	-3.63224100
C	5.19271600	-0.05588800	-4.11756600
N	5.73217400	1.05261100	-3.53892200
C	6.60612700	0.91412300	-2.50245000
C	6.93609800	-0.33154100	-2.00857000
C	-7.11105300	-6.17859100	4.23653200
C	-10.91688000	-0.54597900	-4.22656400
C	-10.37315900	2.16113800	-3.12990500
C	-8.24326200	-3.84819100	5.68378600
C	8.92647500	-3.07600900	4.22049100
C	3.27009600	-10.02541600	-3.14255700
C	7.46644700	-5.20960100	5.67680700
C	-1.80058900	9.23204400	4.23617100
C	4.97982200	9.71313500	-4.22194500
C	7.05191100	7.88696800	-3.12908300
C	0.77811500	9.03774800	5.69232200
C	5.89729000	-9.17222300	-4.23615700
C	5.24812200	-10.49494400	-4.67555800
C	3.71590900	-10.43153900	-4.55765600
C	8.95666400	-3.15482000	5.75641600

C	8.75140800	-4.59444700	6.25727100
C	6.44918700	9.83056100	-4.65906000
C	7.17644500	8.48040000	-4.54300900
C	-1.74753200	9.29268100	5.77210100
C	-0.39990500	9.83772400	6.27460500
C	-11.75301000	0.66844200	-4.66320200
C	-10.94930100	1.97446000	-4.54395300
C	-7.20010300	-6.16171100	5.77213700
C	-8.35006400	-5.26791600	6.26650200
H	-0.38616300	-6.82780400	-3.92071300
H	-0.42335200	-5.41103300	-4.98061800
H	-4.34173900	-3.18293200	-4.08908600
H	-2.15230500	-3.93881400	-4.94938600
H	-1.91651900	-6.99243800	-2.12139600
H	-4.17220700	-6.45442200	-1.22873200
H	-3.94046400	1.23905900	3.11483200
H	-2.12085000	2.93740900	3.19674600
H	-4.59022000	5.88327600	1.58528300
H	-6.52446300	4.30834000	1.44491200
H	-2.49027200	6.40537400	2.17105800
H	-1.72923200	5.40944900	3.40490400
H	0.89408400	-4.03273700	3.15349500
H	-1.49073200	-3.31232900	3.25186600
H	-2.80110500	-6.91267300	1.60953200
H	-0.46960500	-7.79894500	1.47062000
H	-6.37344100	-3.11481600	-3.68720400
H	-5.42934200	-5.26104000	-0.01764300
H	-9.07193700	-2.99501300	-0.35752500
H	-8.27913300	-1.62894600	-3.78983800
H	-8.63679600	-4.13274300	1.66081600
H	-6.13548300	-4.26034100	4.11684900
H	-11.29852100	-0.21432100	-2.13386700
H	-5.97968700	0.56672100	3.97512100
H	-7.22931700	2.40204400	0.22920300
H	-9.19533400	-1.14375700	1.68887000
H	-7.07977800	-1.51592100	4.77925000
H	-9.96152000	-0.17619600	-0.33113800
H	-8.72704100	0.80515300	-3.44719600
H	-9.16901200	-4.43349100	3.82640200
H	-2.42458100	2.05427300	-3.12934300
H	-2.21105000	0.09424200	-1.66701100
H	-6.52200800	-0.08696100	-1.27953900
H	-6.73759200	1.93952500	-2.61918800
H	-5.74988600	3.76333200	-3.89847500

H	-4.51098400	3.08625400	-4.96505400
H	-2.52841200	-2.22266700	-1.55071200
H	-2.15933900	-4.03621000	0.06178000
H	-5.43001200	-2.54306400	2.48043200
H	-5.81005900	-0.74463800	0.84682900
H	-4.30584700	-5.35677400	2.20130900
H	-3.82301900	-4.19143900	3.42805200
H	5.90771400	-3.95761800	-3.70146900
H	7.29239500	-2.07779100	-0.02522100
H	7.13180200	-6.36329700	-0.37028700
H	5.56828400	-6.35066900	-3.80991700
H	7.90150700	-5.42167000	1.65123500
H	6.77643600	-3.18230100	4.10536800
H	5.79905100	-9.67103800	-2.14369500
H	2.50955600	-5.45389700	3.98944300
H	1.52107600	-7.44585500	0.24589500
H	5.58483200	-7.37533900	1.67845100
H	4.86418200	-5.35016300	4.77108200
H	5.11692600	-8.52276700	-0.33921800
H	3.64730700	-7.92366200	-3.44499200
H	8.43379700	-5.72928800	3.82070600
H	-0.54270500	-3.08552400	-3.17131500
H	1.05111400	-1.92850000	-1.70629100
H	3.31376400	-5.59615600	-1.26868400
H	1.66207700	-6.78723100	-2.60933100
H	3.21488100	-1.06643100	-1.56866600
H	4.60987800	0.15086100	0.04240500
H	4.92006100	-3.42513000	2.46935000
H	3.53249000	-4.64195600	0.84429200
H	-0.61164600	5.35554800	-4.09047000
H	-2.36909300	3.84124000	-4.94291500
H	-5.11452900	5.17020300	-2.10091700
H	-3.51400900	6.85068500	-1.21680000
H	3.07557900	2.78734800	3.15518600
H	3.63383100	0.36103500	3.22551800
H	7.40844100	1.03215300	1.58497900
H	7.00746800	3.49311700	1.44653800
H	5.57660800	-1.21897900	3.40689000
H	6.81822400	-1.04890400	2.17309900
H	0.46808000	7.08628100	-3.69286500
H	-1.84746100	7.33208900	-0.01358000
H	1.94106700	9.34306200	-0.35758200
H	2.71484500	7.99008200	-3.79729900
H	0.74301300	9.53180300	1.66670600

H	-0.62782200	7.42697100	4.11596000
H	5.45856800	9.87567000	-2.12899700
H	3.49227200	4.88658200	3.98955200
H	5.70034100	5.04884100	0.24072700
H	3.59699100	8.51939000	1.69130000
H	2.21458100	6.86427800	4.77972900
H	4.81620700	8.69989200	-0.32899600
H	5.05321800	7.13838900	-3.44053000
H	0.74048000	10.14125100	3.83981200
H	2.97732400	1.06955600	-3.16831200
H	1.17786000	1.86185500	-1.69696200
H	3.20005100	5.66813500	-1.27008200
H	5.06098400	4.84180500	-2.61247300
H	6.13249000	3.08702800	-3.91585800
H	4.92687500	2.34775600	-4.97968100
H	-0.65704400	3.29820700	-1.56916900
H	-2.41634300	3.89361500	0.03503200
H	0.51291800	5.95560200	2.47336700
H	2.26685200	5.37065700	0.85141300
H	4.96655200	-2.16123500	-4.11152700
H	4.52993900	0.11631000	-4.96587200
H	7.02337400	1.83910000	-2.10426800
H	7.68314400	-0.38493900	-1.21659700
H	-7.99896900	-6.67189200	3.80075100
H	-6.23497800	-6.75762500	3.89934200
H	-11.50667400	-1.47580500	-4.28102000
H	-10.05820000	-0.67119400	-4.91207600
H	-11.18783800	2.29885300	-2.39522500
H	-9.74421500	3.06464200	-3.07301400
H	-9.07862200	-3.21452200	6.02567900
H	-7.31203900	-3.37476200	6.04686700
H	9.79449400	-3.60504600	3.78652600
H	8.99541400	-2.02993600	3.87794500
H	3.54493600	-10.80753700	-2.41103600
H	2.17437300	-9.91614500	-3.08779200
H	7.33176400	-6.24800800	6.02278800
H	6.59260900	-4.63549200	6.03747400
H	-1.77938600	10.24970500	3.80530000
H	-2.73979500	8.76691400	3.89235700
H	4.46891700	10.68862300	-4.27508700
H	4.44235400	9.03268200	-4.90822100
H	7.58032400	8.52303300	-2.39513100
H	7.51978800	6.89036400	-3.07375000
H	1.74350100	9.44204300	6.03997400

H	0.72058100	7.99259500	6.04960400
H	5.58451400	-8.36146700	-4.92003300
H	6.99684800	-9.22983700	-4.29158200
H	5.63447000	-11.31737200	-4.04489300
H	5.54420500	-10.73231100	-5.71079500
H	3.32296000	-9.69871800	-5.28717100
H	3.26845100	-11.40381800	-4.82224300
H	8.16133400	-2.50655800	6.16988000
H	9.91164100	-2.74938500	6.12907700
H	9.61748700	-5.21650800	5.96396000
H	8.71332600	-4.61509800	7.35880200
H	6.50364100	10.20845200	-5.69340800
H	6.95850700	10.58114600	-4.02613200
H	6.74800600	7.77025500	-5.27505300
H	8.24147100	8.59231600	-4.80529800
H	-1.90740800	8.27747600	6.18145100
H	-2.57838300	9.91254800	6.14723800
H	-0.36320900	9.81232800	7.37604400
H	-0.29756900	10.89991400	5.98403300
H	-12.10571500	0.52799800	-5.69836700
H	-12.65886900	0.73233900	-4.03175200
H	-10.11990100	1.96177700	-5.27581500
H	-11.58035500	2.84022100	-4.80439900
H	-6.24419700	-5.79203100	6.18854700
H	-7.32341100	-7.19113800	6.14673600
H	-8.35405000	-5.22266100	7.36793600
H	-9.31877500	-5.71123900	5.96950500

***P*-*A*-*S*-2**$^{6+}$ (ΔG = 39.4 kcal/mol)
Total SCF energy (BP86-D3/6-311G(d)/PCM(water)): -7021.223077 a.u.
Thermal correction to Gibbs free energy at 298.15 K: 2.486294 a.u.
Gibbs free energy at 298.15 K (BP86-D3/6-311G(d)/PCM(water)): -7018.736783 a.u.

C	2.08199900	-4.92646500	-4.40609500
C	-1.82201800	-5.95629100	-2.90901600
C	-1.61824400	-5.65533600	-4.27818400
C	-0.35088800	-5.37932500	-4.76495900
N	0.73818300	-5.41119900	-3.94721400
C	0.59222300	-5.78419200	-2.64294000
C	-0.65210900	-6.05055000	-2.11345900
C	-4.75665600	0.25101200	2.96459700
C	-3.54885800	-0.03170300	3.64415800
C	-2.65561600	0.97966800	3.93896500

N	-2.90851000	2.27034400	3.59274000
C	-4.10080200	2.59444300	3.02019700
C	-5.03597500	1.62017900	2.72433300
C	-1.79009000	3.27345100	3.58419400
C	4.36730200	-2.64825600	4.28406000
C	3.99451800	-1.85763000	5.39873400
C	2.70318500	-1.90095900	5.90005400
N	1.76162300	-2.71479200	5.34587800
C	2.10780100	-3.55113600	4.32473400
C	3.37699400	-3.53299900	3.78886300
C	-5.54557500	-5.56923900	-2.28582300
C	-4.28889100	-5.52637100	-2.91913500
C	-3.16025100	-6.11415900	-2.31631700
C	-3.31724700	-6.76506700	-1.06866500
C	-4.55647500	-6.80261800	-0.42083600
C	-5.67393400	-6.21684500	-1.04916500
C	-6.69611100	-4.77971100	-2.77323700
C	-4.64955400	-7.33195800	0.95786600
C	-6.28867200	-3.18174600	2.47564100
C	-5.57534900	-2.11307000	3.04470300
C	-5.61645400	-0.83218200	2.45994100
C	-6.40437400	-0.62834600	1.30004900
C	-7.10545900	-1.68914100	0.71507900
C	-7.05485400	-2.95896000	1.32230300
C	-7.83862100	-1.54003700	-0.56866600
C	-1.91038200	-2.20578200	-1.84825600
C	-1.39871000	-2.28880500	-0.55241000
C	-2.24622700	-2.58771400	0.53701200
C	-3.59112000	-2.90761100	0.26698700
C	-4.10665400	-2.81081900	-1.02625800
C	-3.27649700	-2.42200500	-2.09153300
C	-3.88377200	-2.04360200	-3.42269200
C	-1.72977100	-2.52007200	1.92337500
C	-0.76795300	-1.54131000	2.26592000
C	-0.18168500	-1.53437700	3.52940000
C	-0.52861600	-2.50091500	4.49276100
C	-1.56315100	-3.40227800	4.19550800
C	-2.16590500	-3.40434000	2.93018400
C	0.31405000	-2.63538300	5.74758800
C	7.74007200	-1.47266300	-2.38408700
C	6.72799500	-1.17356400	-3.31250900
C	6.27196800	0.15057200	-3.47196200
C	6.85408000	1.18158100	-2.69173200
C	7.83720700	0.88821200	-1.73802500

C	8.28913600	-0.43949900	-1.61016500
C	8.16059300	-2.85689900	-2.08369000
C	8.33732500	1.91999700	-0.79384600
C	7.59756200	-1.19437200	2.93085800
C	6.41139700	-1.34035900	3.67373400
C	5.67835700	-2.54126400	3.62715300
C	6.16933000	-3.61177600	2.84154500
C	7.34492700	-3.48136100	2.09503800
C	8.06210800	-2.26890900	2.15839000
C	8.27706600	0.10921900	2.77438800
C	7.74186100	-4.55643000	1.15884900
C	1.70656700	-2.84779300	-3.00990800
C	2.08271100	-1.97033600	-1.99417900
C	3.35136800	-2.06985500	-1.38337000
C	4.26183100	-3.01466000	-1.89380100
C	3.88058700	-3.90421300	-2.90779300
C	2.58448700	-3.85828300	-3.44837000
C	3.67724500	-1.22034400	-0.21248600
C	2.71373600	-1.00873200	0.79988600
C	2.99201500	-0.17001500	1.87958100
C	4.22982900	0.48922700	1.97758000
C	5.20693200	0.24895700	0.99546400
C	4.93595400	-0.60534300	-0.07754600
C	-4.37291400	2.13219500	-2.62037400
C	-3.30686900	1.65809400	-3.42050300
C	-3.22751300	0.32285700	-3.76459900
N	-4.16674300	-0.57012700	-3.35256800
C	-5.24998000	-0.13712000	-2.64995400
C	-5.38285400	1.19199400	-2.29605200
C	2.04334600	5.00004500	2.26270700
C	2.35555200	4.60913800	3.58604000
C	3.15621200	3.50376900	3.82463800
N	3.66498100	2.76427400	2.79796200
C	3.43446600	3.15298800	1.51427500
C	2.64024300	4.24309900	1.22822600
C	4.45623300	1.50665500	3.06939400
C	-3.52863000	5.79617800	-2.15504200
C	-3.64023600	4.52068700	-2.73290400
C	-4.36690900	3.50284500	-2.08464400
C	-4.99986700	3.78477900	-0.84839100
C	-4.87869900	5.04618800	-0.25430800
C	-4.15384200	6.04986300	-0.92571400
C	-2.65818800	6.84836600	-2.71938700
C	-5.42133300	5.34515700	1.09660700

C	-1.04155700	7.19161500	2.32130800
C	-0.02337500	6.31696900	2.75023100
C	1.09578200	6.07563200	1.93534400
C	1.21401700	6.76395000	0.70624300
C	0.21589700	7.64204200	0.27146500
C	-0.90991300	7.85698000	1.09340200
C	-2.35105700	7.25217900	3.00396600
C	0.28332600	8.18043600	-1.10371100
C	0.32295400	0.85144800	-3.61842900
C	-0.06493800	1.18397600	-2.32178000
C	-0.19634900	2.53477100	-1.92824600
C	-0.06160500	3.53128100	-2.91621600
C	0.33936000	3.19410100	-4.21618100
C	0.58794200	1.85647400	-4.56821100
C	1.26408400	1.49580600	-5.87966500
C	-0.46560800	2.85152400	-0.50721700
C	0.12010000	2.07022400	0.51486800
C	-0.24005400	2.26520600	1.84990500
C	-1.19219700	3.23823200	2.19611300
C	-1.71129700	4.07639900	1.19411400
C	-1.33673300	3.89496600	-0.13765100
C	5.11173200	0.44880900	-4.32807100
C	4.12655600	-0.53396800	-4.58704400
C	2.94121700	-0.19342200	-5.20467300
N	2.67875200	1.09156300	-5.57491100
C	3.64668000	2.04144300	-5.44023400
C	4.86433500	1.74018400	-4.85612700
N	-5.68761400	-7.08560900	1.66429300
C	-5.70843700	-7.40920600	3.09373100
N	-5.07209100	-4.94341900	3.56492600
C	-4.85830500	-6.35796800	3.88088600
C	-5.24145100	-8.82951200	3.47754900
C	-5.00470300	-6.57997000	5.40124700
C	-5.40196800	-9.05832500	4.98971900
C	-4.61346100	-8.01357500	5.79691400
C	-6.13318600	-4.57423700	2.94786000
N	-7.88154900	-2.55426500	-1.34930200
C	-8.62237700	-2.59108600	-2.60518300
N	-6.52094400	-3.75179100	-3.51971800
C	-7.63357100	-2.83924900	-3.79345900
C	-9.54310200	-1.41551300	-2.96847600
C	-8.42537300	-3.22441300	-5.05997700
C	-10.34281200	-1.74874700	-4.24403900
C	-9.42277500	-2.11027800	-5.42336600

N	8.65022100	-4.33804700	0.28463500
C	8.90131100	-5.30359300	-0.78824500
N	7.39929400	-3.85827000	-2.33244200
C	7.77456900	-5.19692500	-1.87081800
C	9.05059700	-6.77735600	-0.35373900
C	8.11632200	-6.08750100	-3.08421300
C	9.40614600	-7.66204400	-1.56004900
C	8.34141400	-7.54939300	-2.66377800
N	8.78298400	1.52783700	0.33992400
C	9.28765400	2.43202600	1.36577700
N	7.65347200	1.21529100	2.95840500
C	8.26750800	2.48430000	2.55256200
C	9.65475300	3.87173600	0.96449000
C	8.89668600	3.21017300	3.76033000
C	10.29715200	4.60752800	2.15483900
C	9.36132400	4.62512300	3.37529600
N	-0.76167100	8.67358800	-1.65310300
C	-0.76363600	8.95341000	-3.09382500
N	-1.66925100	6.55773600	-3.48148400
C	-0.73979800	7.61202600	-3.89938800
C	0.37049100	9.87147200	-3.59775500
C	-0.91183700	7.89308200	-5.40690400
C	0.19962900	10.16186600	-5.09821300
C	0.16737100	8.86186300	-5.91940000
N	-4.77763500	6.18458500	1.81781900
C	-5.20779100	6.64368500	3.13214000
N	-2.74713500	6.29129800	3.75579300
C	-4.14101400	6.23759400	4.20457600
C	-6.60026000	6.25273200	3.65080400
C	-4.39400900	7.01587700	5.51135600
C	-6.89159000	6.99417800	4.97128300
C	-5.81227800	6.72648200	6.03543900
H	1.95524700	-4.55336500	-5.43231800
H	2.76295200	-5.78842400	-4.44708200
H	-2.44768800	-5.64235800	-4.98630200
H	-0.16785300	-5.13010900	-5.81051800
H	1.50384300	-5.81662700	-2.04684300
H	-0.70215300	-6.29487300	-1.05313500
H	-3.25861900	-1.05339700	3.89179500
H	-1.69465400	0.78374500	4.41197600
H	-4.24919900	3.64875000	2.78367700
H	-5.98869100	1.93551100	2.29614000
H	-1.08173800	2.97779300	4.36901900
H	-2.18921500	4.27596600	3.82788200

H	4.71795200	-1.21460700	5.90211300
H	2.38491500	-1.30100800	6.75314200
H	1.31648500	-4.19125800	3.93478000
H	3.57388000	-4.19078200	2.94327700
H	-4.21334600	-4.97982800	-3.86210800
H	-2.46747800	-7.25942400	-0.59016800
H	-6.63294600	-6.23794900	-0.52296800
H	-7.67650300	-5.06489800	-2.35392600
H	-3.75284700	-7.86223400	1.33619100
H	-4.99244200	-2.31301600	3.94674500
H	-6.41598400	0.34871600	0.80705600
H	-7.59646900	-3.78386400	0.85172200
H	-8.32032500	-0.56389000	-0.77626200
H	-1.24824000	-1.93440800	-2.67799500
H	-0.33017600	-2.13195200	-0.38287900
H	-4.25802100	-3.18071700	1.08463400
H	-5.16935300	-3.00845100	-1.19851200
H	-4.82865700	-2.58772000	-3.61678100
H	-3.20444900	-2.20422100	-4.27023600
H	-0.49705500	-0.76755400	1.54091100
H	0.59530300	-0.79925800	3.76062100
H	-1.88382400	-4.13967300	4.93706300
H	-2.96717700	-4.12503100	2.74026400
H	0.06394100	-3.54311100	6.31480900
H	0.21598900	-1.77680500	6.42644900
H	6.32299700	-1.99497900	-3.90797300
H	6.48772400	2.20899800	-2.77197400
H	9.05300000	-0.65650000	-0.85842300
H	9.11311100	-2.94152400	-1.53589600
H	8.28178600	2.97632900	-1.11881600
H	6.05779600	-0.47890100	4.24415400
H	5.64021000	-4.56862900	2.81800700
H	8.96780600	-2.17277300	1.55217000
H	9.30096600	0.05458800	2.37040300
H	7.15489200	-5.49453500	1.22230600
H	0.69752500	-2.78618800	-3.43075300
H	1.39374100	-1.18712600	-1.66786300
H	5.27163400	-3.10758000	-1.48416400
H	4.60605500	-4.65013400	-3.24617700
H	1.75691000	-1.53723600	0.76157600
H	2.23531300	-0.01343800	2.65532100
H	6.18410000	0.73374300	1.06477500
H	5.70005200	-0.76500800	-0.84003700
H	-2.48978700	2.31142400	-3.72929600

H	-2.39152800	-0.08016200	-4.33434600
H	-5.97183000	-0.90158800	-2.36049700
H	-6.28529400	1.49449800	-1.76273300
H	1.98696200	5.17443300	4.44364900
H	3.41859900	3.17886000	4.83151900
H	3.87778000	2.52855500	0.74208000
H	2.43859400	4.46332600	0.18033800
H	4.10418600	1.13479000	4.04358800
H	5.53158100	1.74921900	3.16298100
H	-3.15082200	4.35316900	-3.69527500
H	-5.53182300	2.99452300	-0.30940900
H	-4.05620100	7.02905400	-0.44940800
H	-2.86028400	7.86576400	-2.34617000
H	-6.35914200	4.83897600	1.40144700
H	-0.17021800	5.77364300	3.68697600
H	2.09618100	6.61487500	0.07626300
H	-1.70323200	8.51326200	0.72300800
H	-2.99577000	8.10187300	2.71774200
H	1.24611200	8.02751200	-1.63246100
H	0.45487900	-0.20297400	-3.88116700
H	-0.28980200	0.39143500	-1.60122300
H	-0.24464400	4.58440500	-2.68156500
H	0.48353300	3.99256500	-4.94983600
H	1.29559000	2.33846800	-6.58213100
H	0.78718700	0.65094500	-6.39510600
H	0.87412100	1.31615300	0.26491600
H	0.21214500	1.64240500	2.63034900
H	-2.42862700	4.86242700	1.45066100
H	-1.77269400	4.53518000	-0.90523300
H	4.24685800	-1.56440500	-4.25058100
H	2.15238800	-0.92168600	-5.38509700
H	3.39468900	3.04239500	-5.79151800
H	5.61033900	2.53311700	-4.79083100
H	-6.76204900	-7.30695500	3.41544500
H	-3.79884200	-6.55181400	3.61073200
H	-4.18104700	-8.97174100	3.19892900
H	-5.82512900	-9.56409100	2.89849900
H	-6.05504300	-6.37562700	5.67938300
H	-4.38875300	-5.83718300	5.93494300
H	-5.07024000	-10.07656900	5.25143000
H	-6.47378900	-9.00163900	5.25666700
H	-4.77782900	-8.15612700	6.87770500
H	-3.53034900	-8.15747100	5.62432800
H	-6.92710000	-5.26927800	2.62909000

H	-9.25425200	-3.49767100	-2.51295300
H	-7.15684100	-1.86117700	-4.01000200
H	-8.94305400	-0.49926800	-3.13341300
H	-10.23068800	-1.20309900	-2.13172700
H	-8.95107700	-4.17807200	-4.86918700
H	-7.71930400	-3.40715100	-5.88606100
H	-10.98975700	-0.89756100	-4.51109600
H	-11.01927600	-2.59652200	-4.02911300
H	-10.02370300	-2.41982700	-6.29390500
H	-8.85931800	-1.21288500	-5.74015300
H	9.85412800	-4.99627500	-1.25955400
H	6.85379800	-5.60178100	-1.40004200
H	8.10871400	-7.14014200	0.09731200
H	9.82448600	-6.83999600	0.42910800
H	9.02504700	-5.68012900	-3.56449200
H	7.30681300	-6.00787500	-3.82866600
H	9.51539600	-8.71029600	-1.23637300
H	10.39029900	-7.35555800	-1.96112200
H	8.63242600	-8.14660300	-3.54364900
H	7.38805500	-7.97553800	-2.29873700
H	10.20857100	1.94364400	1.74338200
H	7.42039600	3.10522800	2.19159900
H	8.75106700	4.42067700	0.63886500
H	10.34395900	3.84797300	0.10287000
H	9.74914500	2.60477200	4.11965400
H	8.16519800	3.24443700	4.58435800
H	10.56205200	5.63624800	1.86083000
H	11.24612200	4.10635500	2.42189500
H	9.86178600	5.09664100	4.23694300
H	8.47723900	5.25001200	3.14976600
H	-1.72165200	9.46390200	-3.30640800
H	0.26986100	7.17424800	-3.75226400
H	1.35188400	9.39238500	-3.42513100
H	0.36213900	10.80282800	-3.00795900
H	-1.91968100	8.31951100	-5.56477000
H	-0.88492200	6.93845000	-5.95782600
H	1.01468800	10.81685100	-5.44779400
H	-0.74024900	10.72302800	-5.25636000
H	-0.01072500	9.08324800	-6.98466900
H	1.15585200	8.36852500	-5.86465400
H	-5.17811700	7.74894200	3.04783000
H	-4.32871200	5.16885600	4.43668100
H	-6.65246700	5.15890900	3.81715700
H	-7.36676200	6.49988800	2.89621000

H	-4.26030000	8.09474100	5.31039000
H	-3.63258600	6.73188500	6.25562600
H	-7.88349800	6.70189200	5.35215700
H	-6.94653200	8.07905600	4.76498700
H	-6.00659500	7.33343800	6.93468000
H	-5.86755500	5.67016000	6.35840300
